# Wake‐Riding Effect‐Inspired Opto‐Hydrodynamic Diatombot for Non‐Invasive Trapping and Removal of Nano‐Biothreats

**DOI:** 10.1002/advs.202301365

**Published:** 2023-04-03

**Authors:** Jianyun Xiong, Yang Shi, Ting Pan, Dengyun Lu, Ziyi He, Danning Wang, Xing Li, Guoshuai Zhu, Baojun Li, Hongbao Xin

**Affiliations:** ^1^ Guangdong Provincial Key Laboratory of Nanophotonic Manipulation Institute of Nanophotonics Jinan University Guangzhou 511443 P. R. China

**Keywords:** nano‐biothreats removal, nanorobot, opto‐hydrodynamic effect, opto‐hydrodynamic trapping

## Abstract

Contamination of nano‐biothreats, such as viruses, mycoplasmas, and pathogenic bacteria, is widespread in cell cultures and greatly threatens many cell‐based bio‐analysis and biomanufacturing. However, non‐invasive trapping and removal of such biothreats during cell culturing, particularly many precious cells, is of great challenge. Here, inspired by the wake‐riding effect, a biocompatible opto‐hydrodynamic diatombot (OHD) based on optical trapping navigated rotational diatom (*Phaeodactylum tricornutum* Bohlin) for non‐invasive trapping and removal of nano‐biothreats is reported. Combining the opto‐hydrodynamic effect and optical trapping, this rotational OHD enables the trapping of bio‐targets down to sub‐100 nm. Different nano‐biothreats, such as adenoviruses, pathogenic bacteria, and mycoplasmas, are first demonstrated to be effectively trapped and removed by the OHD, without affecting culturing cells including precious cells such as hippocampal neurons. The removal efficiency is greatly enhanced via reconfigurable OHD array construction. Importantly, these OHDs show remarkable antibacterial capability, and further facilitate targeted gene delivery. This OHD serves as a smart micro‐robotic platform for effective trapping and active removal of nano‐biothreats in bio‐microenvironments, and especially for cell culturing of many precious cells, with great promises for benefiting cell‐based bio‐analysis and biomanufacturing.

## Graphical abstract

Inspired by the wake‐riding effect, this work reports a biocompatible opto‐hydrodynamic diatombot (OHD) based on optical trapping navigated rotational diatom for non‐invasive trapping and removal of nano‐biothreats with sizes down to sub‐100 nm such as adenoviruses, pathogenic bacteria, and mycoplasmas. This OHD can be directly used in cell cultures, without affecting culturing cells including precious cells such as hippocampal neurons.

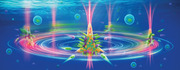

## 1 Introduction

Nanoscale biological threats (nano‐biothreats), such as viruses, mycoplasmas, and pathogenic bacteria, are notorious and adventitious contaminants of cell cultures and bio‐microenvironments. These organisms can modify both the physiology of cultured cells and the structure of recombinant biomolecules. Due to the rapid reproductive capacity, even a small number of nano‐biothreats in bio‐microenvironments can result in a great threat and even a disaster for different biomedical applications ranging from basic cell culturing to bio‐analysis and biomanufacturing.^[^ ^1^ ^]^ Such contamination is widespread in laboratories during cell culturing and can cause great economic losses in biomedical research.^[^ ^2^, ^3^, ^4^ ^]^ Therefore, it is of great urgency to develop efficient tools for nano‐biothreat removal and anti‐bacteria that can be directly used in cell cultures.^[^ ^5^ ^]^ To further facilitate cell‐based biomanufacturing and therapeutics, such tools should be biocompatible and non‐invasive to bio‐microenvironments and cultured cells, which otherwise would generate adverse effects for further biomedical applications such as single‐cell analysis and biomanufacturing. However, the design of effective methods combating the contamination and rapid spreading of nano‐biothreats in bio‐microenvironments and cell cultures, especially during culturing of many precious cells, such as pluripotent stem cells and primary neurons, is always a thorny problem.^[^ ^6^ ^]^

Conventional nano‐biothreat removal methods utilize either chemical (eg., 75% ethanol) or physical (eg., ultraviolet light) agents, which are the most widely used methods for cell cultures.^[^ ^7^, ^8^ ^]^ However, the effective removal and killing of nano‐biothreats requires a high dosage of these sterilization agents, which lacks selectivity and can harm other biological samples, and thus cannot be applied during cell culturing and biomedical application processes.^[^ ^9^, ^10^ ^]^ Although the use of antibiotics has increased the selectivity for bacteria killing, antibiotics increase the risk of bacterial resistance.^[^ ^11^ ^]^ In addition, it is not recommended to use antibiotics during neuron (especially primary neuron) culturing, since antibiotics can affect the living state of neurons. It is also not recommended to use antibiotics when performing gene transfection via cell culturing. Therefore, the design of next‐generation platforms as nano‐biothreat removal and antibacterial agents with high selectivity, efficiency, as well as high biocompatibility, that can actively work during cell cultures, especially for culturing of precious cells, such as pluripotent stem cells and primary neurons, is of great importance and urgency for many biomedical applications.^[^ ^12^, ^13^, ^14^, ^15^ ^]^ Different methods have been developed to meet this requirement. For example, the development of antibacterial nanomaterials has added a new dimension to nano‐biothreat removal and antibacterial action.^[^ ^16^, ^17^, ^18^, ^19^ ^]^ However, many of the nanomaterial‐based platforms are static and the antibacterial action is passive. On the other hand, micromotors (also called microrobots) that can convert external energy into motion hold great potential for controlled navigation and active operation in bio‐microenvironments.^[^ ^20^, ^21^, ^22^, ^23^ ^]^ The dynamic and active feature provides a new suggestion for dynamic antibacterial and active nano‐biothreat removal in bio‐microenvironments. In particular, different strategies based on external energy sources, such as light,^[^ ^24^, ^25^, ^26^ ^]^ magnetic,^[^ ^27^, ^28^ ^]^ and ultrasonic propulsion,^[^ ^29^ ^]^ have been applied for the chemical‐free navigation of micromotors in microenvironments. Among them, magnetic controlled micromotors are widely used for nano‐biothreat removal.^[^ ^30^, ^31^ ^]^ However, all these magnetic‐controlled micromotors necessitate additional specific magnetic materials to respond to magnetic sources for actuation. In another scenario, a micromotor platform directly using microorganisms with self‐propelling capabilities, such as sperm,^[^ ^32^ ^]^ green alga,^[^ ^33^ ^]^ and rotifer,^[^ ^34^ ^]^ can also be used for high‐efficiency nano‐biothreat removal. However, these motile organisms can affect cell growth and metabolism during culturing. In turn, the cell culture media can also affect and destroy the motility feature of the motile organisms, which makes it impossible to further work as a micromotor in cell cultures. Therefore, it is highly desirable to design an intelligent, active, and biocompatible platform that can be directly used in bio‐microenvironments for nano‐biothreat removal. Although optical tweezers (OT) provide huge potential for trapping and manipulating microscopic objects,^[^ ^35^ ^]^ and recently shows the potential for micromotor actuation,^[^ ^36^, ^37^ ^]^ the diffraction limit makes it challenging for trapping nanoscale objects such as the nano‐biothreats. In addition, the strong light intensity needed for stable trapping can induce potential harm to biological samples. On the other hand, hydrodynamic tweezers also show great potential for non‐contact manipulation of micro/nano‐objects via localized microfluidic flow.^[^ ^38^, ^39^ ^]^ However, neither of these two methods can achieve effective nano‐biothreat removal due to their limited range of action.

**Figure 1:**
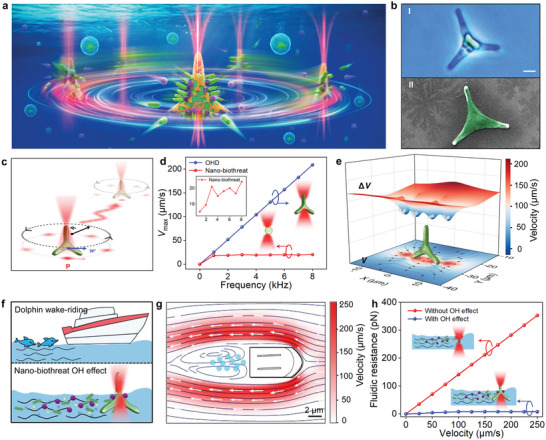
Wake‐riding effect‐inspired OHD. a) Schematic illustration of nano‐biothreat removal in cell culturing microenvironment using OHD. Different nano‐biothreats can be effectively removed, while the viability of the cultured cells is not affected. b) Phase contrast microscope image (bI) and scanning electron microscope image (bII) of PTB. c) Schematic illustration of the motion of the OHD. The black dashed circle with arrows indicates the annular scanning of the trapping laser beam, while the red curved arrow indicates the controlled and targeted navigation of the OHD. d) Calculated maximum flow velocity (*V*_max_) as a function of scanning frequency, inset shows the *V*_max_ of nano‐biothreat in an optical trap. e) Simulated flow velocity (*V*) around the arms of the rotating OHD and relative flow velocity (Δ*V*) to the OHD. Arrows indicate the direction of flow velocity. f) Schematic illustration of the OH effect for nano‐biothreat removal via OHD. g) Simulated flow field of moving microboat with nano‐biothreat behind. h) Fluidic resistance exerted on the nano‐biothreat as a function of forward velocity. Scale bars: 2 µm.

In nature, dolphins often cruise the boat wakes to catch a free ride, allowing them to swim and migrate with far less energy than usual. This phenomenon is called the wake‐riding effect. Such a wake‐riding effect can be found at both low Reynolds numbers (Re) and high Re. For example, in the case of low Re, the wake‐riding effect is utilized for colloidal particles transporting under the assistance of localized flow fields generated by front‐moving particles.^[^ ^40^ ^]^ In the case of high Re, during the process of human swimming, followers are shown to use the leader's wake to offset the wave, thereby reducing the resistance and achieving more efficient swimming.^[^ ^41^ ^]^ Inspired by this wake‐riding effect, in this work, by combing optical trapping and opto‐hydrodynamic effect (OH effect), we report an opto‐hydrodynamic diatombot (OHD) for trapping and removal of nano‐biothreats in bio‐microenvironments of cell cultures based on optical trapping‐navigated triradiate *Phaeodactylum tricornutum* Bohlin (PTB, a widespread diatom in nature) (**Figure** **1**a). It should be noted that we use the term wake‐riding effect because of the similarity between nano‐biothreats trapping by moving OHD and dolphins migrating with boats. Although the wake‐riding effect for dolphins occurs at the water surface, our OHD can work inside fluids. Nano‐biothreats near the wakes of the fast‐rotating OHD are easily trapped via the OH effect, where the hydrodynamic resistance of nano‐biothreats is significantly reduced. This effect greatly increased the nanoscale trapping efficiency of optical tweezers. These OHDs are capable of active and on‐demand removal of different nano‐biothreats in bio‐microenvironments of cell cultures, such as viruses, mycoplasmas, and pathogenic bacteria, without affecting the viability of cultured cells including primary neurons. By coating with chitosan, these OHDs show remarkable antibacterial capability after bacteria collection, without affecting the viability of cultured cells and greatly increasing the survival rate of living cells. These features further facilitate targeted gene delivery. This OHD provides a new method for effective trapping of nano‐objects in the bio‐microenvironment and will serve as a new micro/nano‐robotic platform for non‐invasive, high‐efficiency, and broad‐spectrum removal of nano‐biothreats during cell culturing, including many precious cells, such primary neurons, with great promises for benefiting single‐cell analysis and cell‐based biomanufacturing.

## 2 Results

### 2.1 Experimental Setup for Wake‐Riding Effect‐Inspired OHD for Nanoscale Trapping

Natural PTB exhibits a triradiate morphology with three elongated diatom arms in the normal growth condition (Figure 1b, the average size of the arm: 7.9 µm in length and 1.8 µm in width, average angle 120°, details see Figure S1, Supporting Information). This natural PTB, however, is an immotile diatom and is not able to locomotion. By applying an annularly scanning optical trap (scanning radius *R*: 10 µm, trapping power *P*, scanning frequency *f*) on the PTB (Figure 1c) based on a standard optical tweezers system (Aresis Tweez 250 si, operation wavelength: 1064 nm, details see Figure S2, Supporting Information), an immobile PTB can be turned into a controllably motile OHD (Movie S1, Supporting Information). A typical rotation trajectory of an OHD (Figure S3a, Supporting Information). This rotation speed becomes stable with an optical power larger than 50 mW and a scanning frequency *f* larger than 8 kHz, and stabilizes at a maximum value of about 200 rpm (Figure S3b,c, Supporting Information). However, it should be noted that a large optical power will inevitably cause optical damage and thermal damage to other biological samples in the bio‐microenvironment. Therefore, to get an effective rotation and minimalize the light damage to the biological sample, we selected the parameters of *P* = 50 mW and *f* = 8 kHz to control the OHD in the experiments. The high‐speed rotation of the OHD induces highly localized flow fields and hydrodynamic vortexes around the OHD arms. By moving the OHD along a predefined trajectory of laser trap, the OHD can be navigated to designated locations for targeted nano‐biothreat removal as shown in Figure 1c.

### 2.2 Simulation Analysis

To show the nano‐biothreat removal capability of the OHD, a numerical simulation based on the finite‐element method was carried out. The geometry structure of the OHD in the simulation is shown in Figure S4, Supporting Information, which is in accordance with the structure observed in the SEM images (Figure 1b). Specific simulation details can be seen in Supporting Information. In the simulation, the OHD was subjected to a focused laser beam and locomoted along a predefined circular trajectory (radius *R*: 10 µm). The results show that the maximum velocity of OHD linearly increases with the increase in scanning frequency (*f*) (Figure 1d), whereas the nano‐biothreats tend to be stable after a short acceleration process, due to the limited optical force. This shows that it is very difficult to remove nano‐biothreat by the optical force alone. However, if the nano‐biothreats are placed near the PTB and surf the flow around the PTB, it requires only a small compensation speed (Δ*v*) to follow the PTB (Figure 1e), which can increase the removal efficiency. For the simulation in Figure 1e, the scanning frequency is kept at 8 kHz (the rotation speed of the OHD is 200 rpm), which is the same as that in the experiments. The situation that nano‐biothreats moving along OHD by OH effect is similar to a dolphin riding the wake of a boat to catch a free ride, as shown in Figure 1f. At the microscale, the size difference between OHD and nano‐biothreats is similar to that between a large boat and a dolphin. In both cases, a smaller object utilizes the flow field generated by a larger moving object to catch a free ride and labor‐saving migration with less energy than usual. In order to show the OH effect of OHD on the nano‐biothreats, the flow field distribution of an OHD‐modeled microboat was simulated (Figure 1g), where the forward velocity of the microboat was set the same as the tangential velocity of the rotating OHD (209 µm s^−1^). It should be noted that the vortex behind the microboat in Figure 1g schematically shows the vortex flow behind the microboat. Without the OH effect, fluidic resistance is increased with the increase in forward velocity. For the nano‐biothreat (polystyrene particle, PS, 150 nm) with the forward velocity of 209 µm s^−1^, the fluidic resistance is up to 295 pN. On the contrary, under the OH effect, the calculated maximum fluidic resistance of nano‐biothreat is only 7.9 pN, which is 37 times smaller than that without the OH effect (Figure 1h). Benefiting from the OH effect, a small optical force exerted on the nano‐biothreat can thus overcome this fluidic resistance, resulting in the trapping and collection of the nano‐biothreat by the OHD.

**Figure 2:**
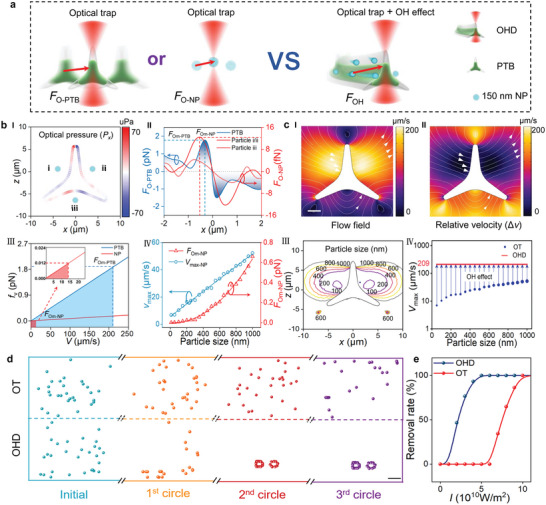
Comparison of nanoscale trapping via sole optical tweezers and OHD with OH effect. a) Schematic illustration of trapping by sole optical tweezers and OHD. b) Numerical calculation of nanoscale trapping by sole optical tweezers. bI) Simulated *x* component of optical pressure on the PTB surface. bII) Calculated optical force exerted on PTB and 150‐nm PS nanoparticle as a function of distance to the focus in the *x* direction. bIII) Fluidic resistance of the PTB and nanoparticle as a function of velocity, inset shows maximum fluidic resistance exerted on the nanoparticle. bIV) Maximum velocity and optical force on nanoparticle as a function of particle size. c) Numerical simulation and calculation of nanoscale trapping via OH effect of OHD. cI) 2D flow velocity generated by moving OHD. cII) 2D relative flow velocity to OHD. cIII) Calculated effective trapping range for particles with different sizes in the flow field generated by the OHD. cIV) Maximum velocity of nanoparticles that can be effectively trapped by OT and OHD. The arrows indicate the velocity transition of different‐sized particles via the OH effect. d) Simulated particle trapping and collection performance using (upper) OT and (lower) OHD. The dots with different colors indicate the particles at different trapping periods. e) Simulated removal rate of OHD and OT as a function of optical intensity. Scale bars: 2 µm.

To further show the OH effect‐assisted trapping of nano‐biothreat by OHD, we analyzed the trapping efficiency of both sole optical trapping and OH‐assisted trapping for 150‐nm PS nanoparticles (**Figure** **2**a). With a focused laser beam irradiated (power: 50 mW), microscopic objects can be trapped by optical force (*F*_O_) via photon momentum and optical pressure change (Figure 2bI). It can be seen that the optical pressure (*P*_x_) on both sides of PTB is equal in value and opposite in direction, resulting in zero resultant optical force. However, as the PTB deviates from the optical axis, the symmetry destruction of the optical pressure generates a spring‐like optical force (*F*_O‐PTB_) that tends to draw the PTB back onto the optical axis (Figure 2bII). The maximum of F_O‐PTB_ (*F*_Om‐PTB_ = 1.99 pN) exists at the distance of 0.28 µm. The optical force (*F*_O‐NP_) exerted on the nanoparticle follows a similar law to PTB, but the value is more than two orders of magnitude smaller, and the maximum value (*F*_Om‐NP_ = 12.4 fN) exists at the distance of 0.6 µm. The results above indicate that the sole optical trapping has a weak confinement ability on nanoparticles. The motion of PTB and nanoparticles in static liquid is restricted by the fluidic resistance (*f*_v_), which is linearly increased with the velocity (*v*) of the object (Figure 2bIII). Calculation results indicate that the maximum velocity (*v*_max_) that can be driven by *F*_Om‐NP_ is 14 µm s^−1^ when trapping of 150‐nm particle, while this *v*_max_ for PTB is 209 µm s^−1^ (Figure 2bIII). Smaller particles move slower in liquids when the optical force and fluidic resistance are balanced during the trapping process. This is because the fluidic resistance is linearly increased with particle size, while the driving force *F*_Om‐NP_ is quadratically increased with an increase in particle size (Figure 2bIV). However, in real experiments, considering the Brownian motion, the instantaneous velocity of random motion for nanoparticles can even be larger than the value of *v*_max_, and thus the particle can escape from the trap. Therefore, for sole optical trapping, it is challenging for effective trapping of nanoscale objects.

In the simulation, we further considered the nanoscale trapping in a moving OHD (*v*_OHD_ = 209 µm s^−1^, same as the calculated *v*_max_ for PTB motion in Figure 2bIII) controlled by optical trapping (Figure 2c). The flow field around the moving OHD in water is depicted in Figure 2cI. Effective removal of nanoparticles requires the particles to follow the moving OHD with the same velocity. However, as previously calculated (Figure 2bIII), nanoparticles cannot travel at this speed by optical force. Fortunately, a localized flow field is generated around the PTB during PTB migration (Figure 2cI). For nanoparticles in such a flow field, a small optical trapping force (*F*_O‐NP_) can overcome this velocity difference, allowing the nanoparticles to remain synchronized with the OHD, which is similar to the wake‐riding effect. Therefore, we are concerned about the velocity difference (Δ*v* = *v* − *v*_OHD_) to OHD as a reference (Figure 2cII). Combining the value of *v*_max_ for different‐sized particles that can be driven by optical force in Figure 2bIV and the contour line of Δ*v* in Figure 2cII, we can obtain the effective capture range of particles with different sizes by the OHD in the flow field (Figure 2cIII). It can be seen that larger particles own a wider capture range. For sole optical trapping, the random motion of nanoparticles that can be suppressed and trapped by optical force is very limited as discussed in Figure 2b. For example, for a 150‐nm nanoparticle, the maximum particle velocity is only 14 µm s^−1^. However, due to the OH effect, the nanoparticles can be confined by the OHD, and rotated together with the OHD (Figure 2cIV), therefore, realizing the transition of trapping/removal velocity (*v*_max_). This efficiency is more obvious for particles with smaller sizes. The Re number of the objects in our system can be estimated according to: Re = *ρvd*/*µ* = 0.01, where *ρ* = 1 × 10^3^ kg m^−3^ is the fluid density, *v* = 1 × 10^−3^ m s^−1^ is the fluid velocity, *d* = 10 µm is the characteristic length, and *µ* = 1 × 10^−3^ Pa s is the dynamic viscosity coefficients. As described by Fox et al., laminar flow exists for the Re number smaller than 2900, while for the Re number larger than 2900, the flow field changes from laminar flow to turbulent flow.^[^ ^42^ ^]^ Therefore, the collection and removal of nano‐biothreats by our OHD are in a laminar flow regime with a low Re number. In addition, we also numerically analyzed the removal rates with the flow fields changing from laminar to turbulent regime by changing the Re number with different dynamic viscosity coefficients. As shown in Figure S5, Supporting Information, with the flow field changing from laminar to the turbulent regime, the removal rate gradually decreases.

To further show the nanoscale trapping and removal capability of the rotating OHD, simulations on multiple 150‐nm PS nanoparticle trapping and collection via both annularly scanning OT and OHD were both performed. In the simulation, the scanning frequency of both methods was kept the same at 8 kHz. As shown in Figure 2d, effective collection is achieved after two circles of OHD rotation, while no obvious collection is achieved even after three circles for OT (details see Movie S2, Supporting Information). This collection performance was also demonstrated experimentally. Effective collection and removal of *Escherichia coli* were realized at an operation time of *t* = 13.5 s for OHD, while 80% of the bacteria were still randomly distributed in the microchannel for OT (Figure S6, Supporting Information). Repeated experiments show that about a 100% removal rate can be achieved after 14 s for OHD, with a much higher efficiency than that of OT at the same time (Figure S7a, Supporting Information). Due to the synergic effect of optical force and hydrodynamic force, this removal capability also depends on the power of the trapping laser. As shown in Figure 2e, the simulated removal rate of OHD increases with the increase in optical intensity, and it is much higher than that for OT under the same optical intensity. From Figure 2e, it can also be seen that the removal becomes stable with the light intensity of 5 × 10^10^ W m^−2^ at the focus. It should be noted that in the simulation, the light intensity shown in Figure 2e is the strongest intensity at the center of the focus. However, in the experiments, the focus size is about 2 µm in radius, and the optical power for effective OHD rotation and nano‐biothreat collection is 50 mW. In this case, the average light intensity at the focus is estimated to be 3.98 × 10^9^ W m^−2^. This light intensity is within the typical light intensity range of optical tweezers, which is in the order of 10^9^ W m^−2^.^[^ ^43^ ^]^ Compared with previously reported microalgae trapping and manipulation with optical power up to 150 mW,^[^ ^36^ ^]^ the power of 50 mW we used for effective nano‐biothreats trapping and collection is relatively low. In the experiments, we find that for optical power less than 40 mW, the removal rate is increased with the increase in optical power, and a removal rate of about 100% can be achieved with an optical power higher than 40 mW for OHD, while OT is only about 11% under the power of 40 mW (Figure S7b, Supporting Information). Compared with other shaped algae or diatoms in nature, such as spherical or spindle‐shaped ones, the most prominent advantage of PTB is the high‐efficiency trapping and collection capability of nano‐biothreats resulting from its triradiate morphology with three elongated diatom arms. For example, for spherical algae (*Chlamydomonas reinhardtii*) and spindle diatom (*Nitzschia Closterium*), 70% and 90% of *E. coli* were still randomly distributed in the microenvironment after 30 s collection under the action of annular optical trap, respectively (Figure S8, Supporting Information), while for our PTB‐based OHD, about 100% of the *E. coli* are trapped and collected. Both the simulation and experimental results show the wake‐riding effect inspired by OHD exhibits a remarkable removal capability due to the synergic opto‐hydrodynamic force and thus can be used for nano‐biothreat trapping and removal.

### 2.3 Non‐Invasive Trapping and Removal of Nano‐Biothreats

**Figure 3:**
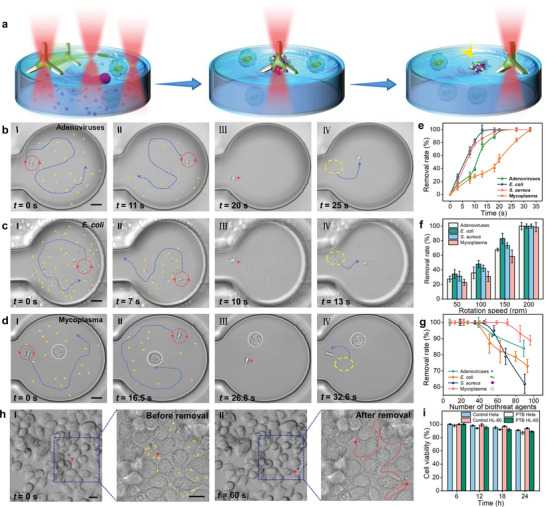
Non‐invasive trapping and removal of nano‐biothreats. a) Schematic illustration of trapping and removal of nano‐biothreats in a microfluidic channel via OHD, yellow arrows indicate released nano‐biothreats. b–d) Trapping and removal of b) adenoviruses, c) *Escherichia coli*, and d) mycoplasmas. Panels I–IV: microscopic images show the trapping and removal at different times, yellow arrows indicate the nano‐biothreats, red dashed circles and blue curves in panels I and II indicate the rotational position and navigation trajectory of the OHD, respectively. Yellow dashed circle and blue arrow in panel IV indicate the released nano‐biothreats and navigation of the OHD after nano‐biothreats release, respectively. White dashed circle in (d) indicates the cultured HL‐60 cell. Scale bars: 3 µm. e) Final removal rate as a function of time. f) Final removal rate as a function of rotational speed. g) Final removal rate as a function of nano‐biothreat number. h) Removal of invaded *E. coli* bacteria in real dense cell cultures with cell confluency of about 90%. I) Before removal, II) after removal. Red arrow indicates the OHD, yellow arrows indicate the invaded bacteria, and red curve indicates the working trajectory of the diatom during bacterial removal. Scale bars: 30 µm. i) Cell viability as a function of time for different treatments.

As the three most widely existing nano‐biothreats in cell cultures, the contamination of viruses, pathogenic bacteria, and mycoplasmas are great threats to many cell‐based biomanufacturing and therapeutics. To show the non‐invasive nano‐biothreat trapping and removal capability of our OHD, a demonstration of nano‐biothreat trapping and removal in a microfluidic channel was carried out (**Figure** **3**a). Randomly distributed nano‐biothreats are first trapped and collected around the OHD arms during OHD rotation (200 rpm). The collected nano‐biothreats can be swept to a designated location via navigating the OHD. By turning off the trapping laser, the localized flow fields around the OHD arms disappear, and the collected nano‐biothreats can be released at the designated location. Subsequently, the annular scanning optical trap is switched to a central trap with lower power (5 mW), and the OHD can be navigated to other positions for repeated use in subsequent experiments.

As some examples, Figure 3b–d shows the trapping and removal of adenoviruses (90–100 nm in diameter),^[^ ^44^ ^]^ pathogenic bacteria (rod‐shaped gram‐negative *E. coli*), and mycoplasmas by using our OHD (*P* = 50 mW and *f* = 8 kHz) in a disc‐shaped microfluidic channel (diameter: 100 µm, depth: 50 µm, fabrication details, see Experimental Section). As shown in Figure 3b, about 20 randomly distributed adenoviruses were completely trapped by the OHD at *t* = 20 s. By turning off the laser, the collected adenoviruses were released from the OHD and completely removed from the channel at *t* = 25 s, and the OHD was then moved to another position. Due to the small size of nano‐biothreats, such as the virus and bacteria, and the diffraction limit, it is difficult to clearly observe the nano‐biothreats. Therefore, we assigned yellow arrows at the positions of nano‐biothreats for better identification. To avoid confusion caused by the yellow arrows, Figure S9, Supporting Information, shows a representative raw image, as well as that with the yellow arrow assigned during virus collection. Details of the trapping and removal of adenoviruses are shown in Movie S3, Supporting Information. To show the trapping and removal capacity of the OHD for different‐sized nano‐biothreats, PS particles with different sizes were used as the nano‐biothreat models for demonstration. Experimental results show that our OHD is capable of the effective trapping and removal of PS particles with sizes from 100 nm to 2 µm (Figures S10–S12, Supporting Information). In addition to the effective removal of immotile abiotic particles that imitate nano‐biothreats, importantly, our OHD can also be used for the effective removal of motile nano‐biothreats, for example, pathogenic bacteria. Pathogenic bacteria are a common contamination during cell cultures. For example, contamination of a small number of pathogenic *E. coli* can result in the death of both HeLa cells and human promyelocytic leukemia cell line HL‐60 within 12 h (Figure S13, Supporting Information), due to the rapid bacterial reproduction in the cell culture medium. Effective removal of rod‐shaped *E. coli* and spherical *Staphylococcus aureus* were realized, respectively (Figure 3c; details see Figures S14 and S15 and Movie S4, Supporting Information). In addition, in order to more clearly demonstrate the removal process of nano‐biothreats, as an example, we performed additional experiments using fluorescent *E. coli*. The collection and removal process can be clearly observed under a fluorescent microscope (Figure S16, Supporting Information).

In addition to the effective trapping and removal capability, the non‐invasiveness and biocompatibility of the OHD are also very important for further cell‐based biomedical applications. To show the non‐invasiveness of the OHD during nano‐biothreat removal, we further carried out experiments on the removal of mycoplasmas in the channel containing both mycoplasmas and cultured mammalian cells (HL‐60). Mycoplasma is one of the most common contaminants during cell culture and can result in the destruction of healthy cells (Figure S17, Supporting Information). Because of the small size and deformability, it is difficult to remove mycoplasma efficiently by traditional filtration methods. By using our OHD, it is capable of highly efficient and selective removal of mycoplasmas. As shown in Figure 3d, about 19 mycoplasmas were collected and removed at *t* = 32.6 s. Importantly, during the mycoplasmas collection and removal, the HL‐60 cell can be avoided from the affection of the OHD, and it was kept intact during the mycoplasma removal process (details see Figure S18 and Movie S5, Supporting Information). This indicates that the OHD exhibits a non‐invasive feature for nano‐biothreat removal. The effective removal time of a single OHD for different nano‐biothreats is different (Figure 3e). In addition, the removal efficiency of OHD for different nano‐biothreats is also related to its rotation speed. As shown in Figure 3f, when the rotation speed reaches 200 rpm, the removal rate can reach the best of 100%. The collection and removal capacity is different for different nano‐biothreats. As shown in Figure 3g, for the nano‐biothreats we used, the saturation removal numbers of adenoviruses, *E. coli*, *S. aureus*, and mycoplasmas were about 39, 40, 45, and 71 with a single OHD, respectively.

Importantly, our OHD can work in both very fluid microfluidic environments and really dense cell cultures. Our OHD can go into the dense cell cultures through the narrow gaps between neighboring cells to remove the invaded nano‐biothreats. To show the applicability of our OHD for nano‐biothreats trapping and removal in dense cell cultures, we have performed additional experiments in bacteria‐contaminated real dense cell cultures. As an example, Figure 3h shows the trapping and removal of contaminated *E. coli* in real dense cell cultures (HeLa cells, cultured in a real petri dish with cell confluency of up to 90%). In these highly dense cell cultures, the OHD can also easily trap and remove the contaminated *E. coli*. As recommended in a cell culture guide posted in BiteSize Bio (https://bitesizebio.com/63887/cell‐confluency/), the ideal cell confluency is 70–80% for real cell culture, and cells should be split at this confluency stage to improve the overall cell viability. These results demonstrate that our OHD can work in real dense cell cultures with cell confluency even higher than the ideal cell confluency for cell culture. After the trapping and collection by the OHD in a petri dish containing the cell culture, we can move the OHD with collected nano‐biothreats to a designated position by optical tweezers, and then both the OHD and the collected nano‐biothreats can be extracted outside from the cell culture media using a pipette. To show the biocompatibility of the OHD to the confluent cells, we have also performed additional experiments for cell viability tests in confluent cells treated with OHD. After culturing confluent cells with 24‐h OHD treatment, as shown in Figure S19, Supporting Information, the viability of the cells was not affected, which was similar to cells without OHD treatment.

To further show the non‐invasiveness and biocompatibility feature of the OHD, we tested the biocompatibility of OHD to two different mammalian cell lines (adherent HeLa cells and suspending HL‐60 cells). After co‐culturing OHD with the cells for 24 h, both cell lines show no obvious decrease in cell viability (Figure S20, Supporting Information, green fluorescence for living cells, blue fluorescence for nuclei). The cell viability is not affected by OHD, which is similar to that of normal cell culturing (Figure 3i). These results indicate that the PTB‐based OHD is highly biocompatible with bio‐microenvironments and mammalian cells.

### 2.4 OHD Array for Efficiency‐Enhanced Removal in Cell Cultures

**Figure 4:**
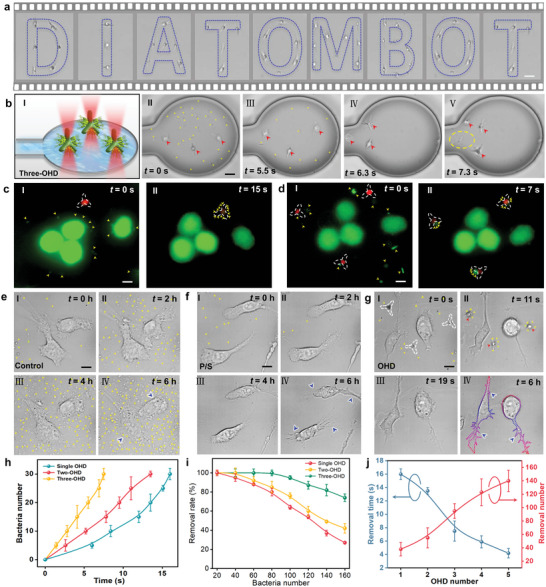
OHD array for efficiency‐enhanced removal. a) Microscopic images showing formed OHD arrays with patterns of “D,” “I,” “A,” “T,” “O,” “M,” “B,” “O,” and “T” in the microfluidic channel, representing “diatombot.” b) *Escherichia coli* removal via a single OHD. Panel (I) shows the schematic for removal, panels (II–V) show microscopic images of removal at different times. The yellow and red arrows indicate *E. coli* and OHD, respectively. The yellow dashed circle indicates the removed and released *E. coli*. c,d) Fluorescence images showing the removal of *E. coli* in HL‐60 cells by c) a single OHD and d) a three‐OHD array. Live HL‐60 cells and live *E. coli* are in green, white dashed triradiate structures indicating OHD, and yellow arrows indicate *E. coli*. e–g) Microscopic images showing the living states of *E. coli* contaminated primary hippocampal neurons with different treatments of e) no treatment, f) 1% pen/strep treatment, and g) OHD array treatment. Yellow arrows indicate *E. coli*, blue arrows indicate the boundaries of neurons after culturing of 6 h, and red arrows indicate OHD. h) Collected bacteria number as a function of time for different OHD arrays. i) Final removal rate as a function of bacteria number in the channel. j) Maximum removal time (left axis, blue) and removal number (right axis, red) as a function of OHD number in the array. Scale bars: 5 µm.

Although single OHD is capable of non‐invasive nano‐biothreat trapping and removal, the efficiency is limited by individual operation. As the number of nano‐biothreat increases, the collection and removal capacity can be saturated, and the removal rate of the OHD is gradually decreased when exceeding the saturation number (Figure S21, Supporting Information). Therefore, the formation of OHD arrays with highly reconfigurable and controllable capabilities is important for efficiency‐enhanced multi‐task execution and manipulation, with higher speed and larger collection volume. Fortunately, our OHD can be extended into OHD arrays with high reconfigurability and controllability, and multiple OHDs can operate sequentially or simultaneously. By extending the single optical trap into trap arrays, multiple traps with designated patterns can be formed. Multiple PTB cells can then be turned into OHD arrays. The rotation of each OHD element in the array can be controlled similarly to that of a single OHD. As some examples, **Figure** **4**a shows the formed OHD arrays with the pattern of “DIATOMBOT.” These OHD arrays with designated patterns will provide more choices for cooperative robotic operation and on‐demand task execution with higher efficiency. These OHD arrays can work independently and collaboratively for nano‐biothreat removal with higher efficiency than that for a single OHD. For a single OHD, the trapping of 25 *E. coli* was completed at 14 s (Figure S22, Supporting Information). The completion collection time was reduced to 7.3 s and 6.3 s for a two‐OHD and three‐OHD array, respectively (Figure 4b; details see Figure S22 and Movie S6, Supporting Information). The efficiency for a three‐OHD array is more than twice that for a single OHD. A comparison of the collection and removal for a single OHD and OHD arrays are also shown in Figure 4h.

These OHD arrays can be directly used in cell cultures for high‐efficiency nano‐biothreat removal without affecting the cultured cells. As shown in Figure 4c, for a single OHD, the removal of 14 *E. coli* in cultured HL‐60 cells was completed within 15 s. For a three‐OHD array, the removal efficiency was more than twice that for a single OHD. Removal of a similar number of *E. coli* with a three‐OHD array was completed within only 7 s (Figure 4d). During the removal, the viability of the cultured cells was not affected. This capability is also applicable to precious cells, such as hippocampal neurons, which are very difficult to extract and cannot be passaged. As shown in Figure 4e, during the in vitro culturing of hippocampal neurons with a small amount of *E. coli* contamination, owing to the fast‐multiplying ability of bacteria, the neurons were totally invaded and even lysed by the bacteria after 6 h. Although antibiotic treatment can effectively inhibit bacteria proliferation during cell culturing, antibiotics can affect the living state of neurons and induce irreversible damage to the neurons, and therefore it is not recommended to use antibiotics during neuron (especially hippocampal neurons) culturing. As shown in Figure 4f, with the treatment of 1% penicillin/streptomycin (pen/strep), although an effective inhibition of bacterial proliferation was achieved, the neurons were irreversibly damaged by the antibiotics, leading to a shrinking in the cell body and inhibition of neurite outgrowth after cultured for 6 h. With the OHD array for bacterial removal, as shown in Figure 4g, the contaminated *E. coli* were effectively collected and removed in only 19 s. Importantly, after the removal, the cultured neurons remained in a good living state, as evidenced by the outgrowth of a higher number and longer neurites. Importantly, after the removal, the cultured neurons remained in a good living state with new branches growing out. These results indicate that our OHD can be directly used for biothreat removal during neuron cell culturing. Due to the collection capacity limit of a single OHD, the total removal capacity for different OHD arrays is also different, and this capacity is increased with the increase in OHD number in the array (Figure 4i,j). For bacteria number that exceeds the removal capacity of a single OHD, although a single OHD cannot completely remove the bacteria, OHD arrays can get a perfect removal efficiency (Figure 4i and Figure S23, Supporting Information). For a given number of *E. coli*, the removal time is decreased with the increase in the number of OHD in the array (Figure 4j). These results indicate that we can build OHD arrays with more OHDs to further get a higher removal efficiency and capacity.

### 2.5 Non‐Invasive Antibacterial Capability for Enhanced Gene Delivery

**Figure 5:**
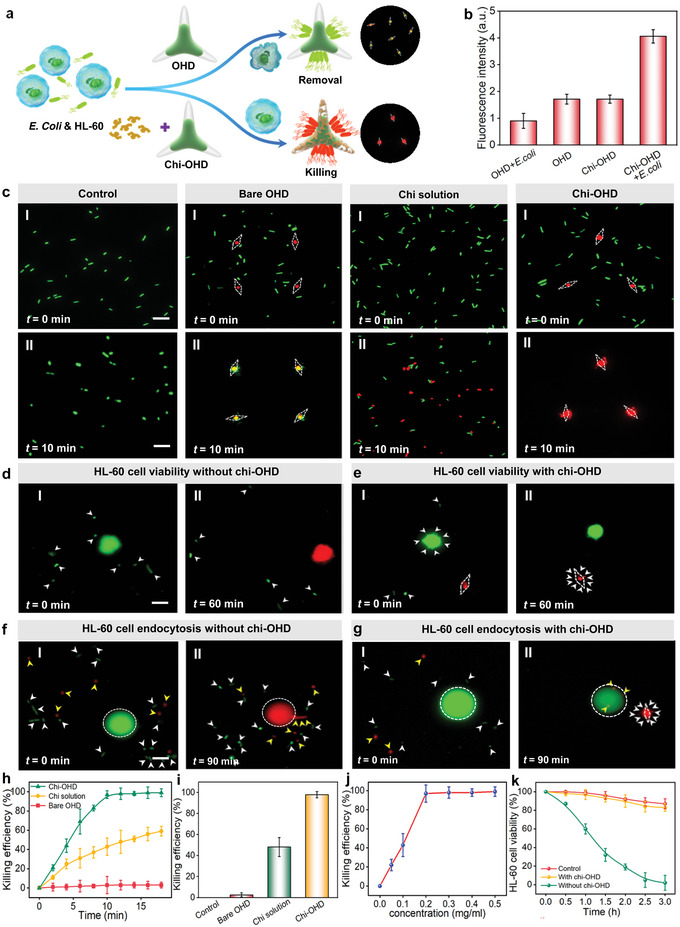
Chi‐OHD for non‐invasive antibacterial performance and enhanced gene delivery. a) Schematic illustration of non‐invasive antibacterial performance by chi‐OHD. Upper inset: fluorescent image showing live *Escherichia coli* and PTB with yellow fluorescence, lower inset: fluorescent image showing dead *E. coli* with red fluorescence after being treated with chi‐OHD. b) Fluorescence intensity of bare OHD, bare OHD with *E. coli*, chi‐OHD, and chi‐OHD with *E. coli*. c) Fluorescent image showing the killing of *E. coli* with different treatments, live *E. coli* in green and dead *E. coli* in red. White dashed rhombuses indicate the position of the OHD. Upper: before treatment, lower: after treatment with 10 min. d,e) Fluorescent image showing the viability of HL‐60 cells contaminated with *E. coli*: d) without treatment of chi‐OHD, e) with treatment of chi‐OHD. White arrows indicate *E. coli*. f,g) Fluorescent image showing gene delivery for contaminated HL‐60 cells treated f) without chi‐OHD and g) with chi‐OHD. White dashed circle indicates the boundary of the HL‐60 cell, and yellow arrows indicate the siRNA‐loaded MSNs. h) Antibacterial efficiency as a function of time for different treatments. i) Antibacterial efficiency for different treatments. j) Antibacterial efficiency as a function of chitosan concentration for OHD coating. k) HL‐60 cell viability as a function of time with different treatments. Scale bars: 5 µm.

Despite the non‐invasive and high‐efficiency nano‐biothreat collection and removal capability of OHD in bio‐microenvironments, the further non‐invasive and efficient killing of contaminated nano‐biothreats in the bio‐microenvironments are very important to ensure further single‐cell analyses. Importantly, in addition to the nano‐biothreat removal, our OHD is also capable of non‐invasive bacterial killing and antibacterial treatment. As shown in **Figure** **5**a, the OHD is modified with a chitosan (Chi) layer as a micro‐robotic strategy for antibacterial treatment in cell culturing microenvironments. Chitosan has garnered increasing interest in the field of antibacterial as a renewable material due to its unique properties such as high biocompatibility, ease of decomposition, and low toxicity.^[^ ^45^ ^]^ Our antibacterial strategy relies on the combination of the efficient nano‐biothreat removal capability of OHD and the strong bactericidal activity of the chitosan layer. Taking *E. coli* as an example, naked OHD can realize *E. coli* removal. Nevertheless, it does not exhibit antibacterial properties, and there still exists an infection risk for the cultured cells. In this case, the final fluorescence of the OHD with collected live *E. coli* stained with a bacterial viability kit (details, see Experimental Section) is yellow. However, by coating a layer of chitosan, the collected *E. coli* can be killed by the OHD, demonstrating the good antibacterial effect of OHD (Figure S24, Supporting Information). Therefore, there is no infection risk for cultured cells. Since the OHD is fluorescent red and the dead *E. coli* also has red fluorescence, the measured fluorescence intensity also reflects the enhanced antibacterial effect of the chitosan‐coated OHD (chi‐OHD, Figure 5b).

In order to show the antibacterial ability of the chi‐OHD, we carried out a series of different controlled experiments. To show the bacterial viability after different treatments, a commercial viability kit based on two dyes (DMAO: 9‐Octadecen‐1‐amine,*N*,*N*‐dimethyl‐,(9Z)‐ and EthD‐3: Ethidium Homodimer 3) was used for *E. coli* staining. The DMAO dye (green) was used to label live bacteria, while the EthD‐3 dye (red) could only penetrate damaged bacteria and was used to label dead *E. coli*. As shown in Figure 5c, after the collection of *E. coli* for 10 min, the bare OHD fluoresces yellow, which is the combination of red (for PTB) and green (for live *E. coli*) fluorescence. This phenomenon indicates that bare OHD cannot result in *E. coli* killing. For the treatment with chitosan only, the fluorescence of about 40% *E. coli* is red, while the others are still green after 10 min treatment, indicating only about 40% of the bacteria are killed by the chitosan solution. However, for chi‐OHD, all the fluorescence is red, indicating all the bacteria are killed after 10 min. The high antibacterial efficiency obtained by the chi‐OHD reflects the key role of the combination of chitosan and the effective rotation of OHD for antibacterial activity. The OHD rotation increases chitosan‐bacteria interaction, and thus results in a high‐efficiency antibacterial performance. Figure 5h,i show the comparison of the antibacterial efficiency for naked OHD, chitosan solution, and chi‐OHD. This antibacterial performance is dependent on the concentration of the chitosan solution for OHD coating. When the chitosan concentration reaches 0.2 mg mL^−1^, the antibacterial efficiency of the OHD reaches 98% (Figure 5j).

This antibacterial capability is of great importance for cell culturing and further for single‐cell analysis and cell‐based biomanufacturing. As shown in Figure 5d, for HL‐60 cells contaminated with *E. coli*, without any treatment, once the living HL‐60 cells (green) are infected by active bacteria, the cell is dead after 60 min (red). However, by using chi‐OHD for bacteria removal and antibacterial treatment, the HL‐60 cell is not infected by bacteria even though the microenvironment is contaminated with *E. coli*, and the cell viability is not affected after 60 min (green fluorescence, Figure 5e). Although the chi‐OHD can kill bacteria, chi‐OHD is highly biocompatible and non‐invasive to the cultured cells (Figure S25, Supporting Information). This non‐invasive antibacterial feature further facilitates the study of enhanced drug delivery and single‐cell‐based therapy. To show this capability, Cy3‐labeled small interfering RNA mimics (siRNAs) that fluoresce red were loaded into mesoporous silica particles (details, see Experimental Section) and added to the cultured HL‐60 cells contaminated with *E. coli*. The bacteria contamination and infection result in cell death (red fluorescence), and siRNA cannot be delivered into the HL‐60 cells (Figure 5f). However, with the treatment of chi‐OHD, the contaminated *E. coli* are completely removed and killed. The cell viability is thus not affected by the contaminated *E. coli* even after 2 h, which is similar to the cells without any contamination (Figure 5k). Since the cell viability is not affected by the contaminated *E. coli*, the siRNA is thus delivered into the cell in 90 min (Figure 5g). These results indicate that the non‐invasive antibacterial capability of chi‐OHD can be directly used to remove and kill the contaminated nano‐biothreats in cell cultures, and further for enhanced drug delivery and subsequent single‐cell analysis.

## 3 Conclusion

In summary, inspired by the wake‐riding effect, we created a non‐invasive and active OHD based on a natural diatom for nano‐biothreat removal, which can be directly used in cell cultures for enhanced gene delivery. The synergic optical trapping and hydrodynamic force of OHD results in high‐efficiency trapping and removal of different nano‐biothreats, including adenoviruses, pathogenic bacteria, and mycoplasmas, in cellular environments without affecting culturing cells including precious primary neurons. This removal capability was further enhanced by reconfigurable OHD array formation. Efficient and non‐invasive antibacterial performance was demonstrated in a cellular environment with chitosan‐coated OHD, which further facilitated single‐cell gene delivery.

This OHD provides a new method for the effective trapping of nano‐objects in bio‐microenvironment, and serves as a smart micro‐robotic platform for non‐invasive and active nano‐biothreat removal and antibacterial treatment in cellular environments, offers a seamless interface among optical, microrobotic, and biological worlds. Due to the non‐invasiveness and high biocompatibility, this OHD can be directly used in cell cultures for the removal of nano‐biothreat contamination, without affecting the cultured cells including precious cells. This feature ensures subsequent cell‐based biomanufacturing and bio‐analysis after nano‐biothreats removal and antibacterial treatment. OHD arrays ensure the capability of larger‐scale removal of nano‐biothreats via the cooperation of microrobot individuals. This OHD provides a biocompatible and smart platform for non‐invasive, high‐efficiency, and broad‐spectrum removal of nano‐biothreats, especially the rapid spreading drug‐resistant bacteria, in bio‐microenvironments of cell culturing of precious cells, such as neurons, with great promises for benefiting cell‐based biomanufacturing and single‐cell analysis.

## 4 Experimental Section

### Experimental Setup of Optical Tweezers

A scanning optical tweezers system (Aresis, Tweez 250Si, Section S3, Supporting Information) was built on an inverted optical microscope (Nikon Eclipse Ti‐U) using a continuous wave solid‐state laser with 1064‐nm operation wavelength (maximum output power: 5 W). The laser beam was refocused into the sample chamber after passing through a 60× water immersion inverted objective (numerical aperture: 1.0) for optical manipulation. Multiple trap sequences could be constructed in a loop manner by a computer‐interfaced AOD system, and the high switching rate (maximum 100 kHz) ensured stable capture and precise manipulation of multiple targets at the same time. The real‐time image was recorded by a computer‐interfaced high‐speed CCD camera. Details of the setup can be seen in Figure S2, Supporting Information.

### PTB Preparation and OHD Construction

PTB diatom solution was purchased from Zhuhai Xinrui Trading Co., Ltd, China. PTB cells were obtained through a 47 mm × 5 µm mixed cellulose ester filter, and the PTB suspension was then prepared by resuspending PTB cells into 1 mL phosphate‐buffered saline (PBS) buffer, with a final concentration of about ≈2 × 10^4^–4 × 10^4^ cell/mL. This PTB solution was then transferred to the sample slide on the scanning optical tweezers system. Next, a central optical trap was applied to trap the PTB, and an annular scanning optical trap controlled by the AOD system was applied to the PTB for rotation and OHD construction.

### Microfluidic Channel Fabrication

Silicon elastomer and curing agent were mixed at a weight ratio of 10:1 for channel fabrication. The solution was placed in a vacuum pump to remove air bubbles that were generated during the mixing process. 50‐µm thick SU8‐3005 photoresist was coated on a clean Si/SiO_2_ wafer and exposed to a standard lithography setup to prepare the master mold of the microfluidic channel. The bubble‐free polydimethylsiloxane (PDMS) was cast over the SU8 photoresist master mold and cured at 70 °C for 3 h. After the curing process, the PDMS was peeled off from the mold and bonded to the glass slide by using O_2_ plasma bonding.

### Nano‐Biothreat Preparation

Adenoviruses were purchased from Guangzhou VectorBuilder Co., Ltd., China, and were suspended in a carbonate buffer (pH = 9.3). Adenovirus suspension with a final concentration of about ≈2 × 10^3^–6 × 10^3^ per microliter was prepared. The bacteria (*E. coli* and *S. aureus*) were grown for 3–4 h in lysogeny broth in a shaker with a rotation speed of 180 rpm at 37 °C. The bacteria were washed and diluted with PBS buffer to obtain a suitable concentration of about ≈2 × 10^4^–5 × 10^4^ bacteria per microliter. Mycoplasma‐contaminated cell culture suspension (1 mL, containing HL‐60 cells) mixed with PTB suspension. After preparation, the suspension and PTB mixture were injected into the microfluidic chamber by a microsyringe for the following experiments. 4“,6‐Diamidino‐2”‐phenylindole (DAPI) was used to stain the cells contaminated with mycoplasma.

### Cell Culturing and Viability Test

Mammalian cell lines (HeLa cells and HL‐60 cells) and PTB cells were co‐cultured in Dulbecco's Modified Eagle Medium (DMEM) supplemented with 4500 mg L^−1^, 10% fetal bovine serum (FBS), and 1% Pen/strep, and then mixed overnight in a 37 °C incubator contained 5% CO_2_. Mammalian cell viability was tested using dual‐fluorescent calcein‐AM/propidium iodide (PI) (purchased from Jiangsu KeyGEN BioTECH Corp., LTD, China). In the experiments, 2‐µL AM and PI dyes were added to co‐cultured cells for 25 min. For living cells, Calcein‐AM can react with esterases, and then strong green fluorescence was emitted for live cells. PI cannot pass the living cell membrane but can reach the nucleus through the disordered region of the dead cell membrane. Therefore, the DNA double helix structure in the cell was bound with PI, so red fluorescence was emitted for dead cells.

### Chi‐OHD Preparation

Chitosan was dissolved in 2% glacial acetic acid to prepare a chitosan solution. In the experiments, 1 mg mL^−1^ chitosan solution was mixed with OHD, and then the chi‐OHD suspension was placed on a shaker with a rotation speed of 200 rpm to shake at 37 °C for ≈3–4 h. Finally, the chi‐OHD suspension was centrifuged for 10 min at a rotation speed of 1500 rpm, and the supernatant was removed to get the chi‐OHD precipitation. The antibacterial ability of the chi‐OHD was tested by analyzing the activity of *E. coli* cells mixed with the chi‐OHD precipitation. Living *E. coli* were collected by chi‐OHD through a standard optical tweezers system. Then the mixture was reacted for 10 min, and the *E. coli* were killed. Bacterial viability kit (DMAO: 9‐Octadecen‐1‐amine,*N*,*N*‐dimethyl‐,(9Z)‐; and EthD‐3: Ethidium Homodimer 3 dyes) (purchased from Yeasen Biotech Co. Ltd. Shanghai, China) was used to test the viability of *E. coli* collected by chi‐OHD and OHD. 2 µL of DMAO and EthD‐3 dye were added to 100 µL of the mixture and incubated with *E. coli* for 20 min for viability characterization.

### Preparation of siRNA‐Loaded Mesoporous Silica Particles

Cy3‐labeled siRNAs (sequence: 5′‐UUCUCCGAACGUGUCACGUTT) were obtained from GenePharma Co. Ltd. (Shanghai, China). Mesoporous silica particles (MSNs) with a diameter of 1 µm were obtained from Wuhan Huake Microtechnology Co., Ltd, Shanghai, China. For the loading of siRNAs, 500 µg MSN was dissolved in 500 µL diethyl pyrocarbonate (DEPC), with a weight concentration of 10 µg µL^−1^. Then, 50 µL siRNAs were added to 200 µL MSN solution and stirred for 24 h. Thereafter, the siRNA‐loaded MSNs were centrifuged and washed twice with DEPC solution and then freeze‐dried for 6 h.

### Nano‐Biothreats Tracking in Supporting Movie

First, the process of nano‐biothreats trapping and collection was captured via a computer‐interfaced high‐speed CCD camera in a video format with 60 frames per second. Then, the video was analyzed frame by frame, and yellow arrows were then assigned to the positions of the nano‐biothreats at each individual frame image. Finally, all of the frame images with the yellow arrow assigned were combined into a new video.

## Conflict of Interest

The authors declare no conflict of interest.

## Author Contributions

J.X. and Y.S. contributed equally to this work. J.X. and H.X. conceived the idea and designed the experiments. Y.S. carried out the simulation. J.X., T.P., and D.L. performed the experiments. Z.H. reviewed and revised the original draft. D.W., X.L., and G.Z. discussed the manuscript. H.X. and B.L. supervised the study.

## Supporting information

Supporting Information

Supplemental Movie 1

Supplemental Movie 2

Supplemental Movie 3

Supplemental Movie 4

Supplemental Movie 5

Supplemental Movie 6

## Data Availability

The data that support the findings of this study are available from the corresponding author upon reasonable request.
