# Supplementary material for: Wake‐Riding Effect‐Inspired Opto‐Hydrodynamic Diatombot for Non‐Invasive Trapping and Removal of Nano‐Biothreats
Source: Adv Sci (Weinh). 2023 Apr 3;10(18):2301365. doi: 10.1002/advs.202301365 (PMC10288256; doi:10.1002/advs.202301365)
Supplement: Supplementary file 1 — Supporting Information [file ADVS-10-2301365-s007.pdf]

ADVANCED SCIENCE
Open Access

Supporting Information

for *Adv. Sci.*, DOI 10.1002/advs.202301365

Wake-Riding Effect-Inspired Opto-Hydrodynamic Diatombot for Non-Invasive Trapping and Removal of Nano-Biothreats

Jianyun Xiong, Yang Shi, Ting Pan, Dengyun Lu, Ziyi He, Danning Wang, Xing Li, Guoshuai Zhu, Baojun Li and Hongbao Xin**

Supporting Information

Wake-riding effect inspired opto-hydrodynamic diatombot for non-invasive trapping and removal of nano-biothreats

Jianyun Xiong[1], *Yang Shi*[1], *Ting Pan*[1], *Dengyun Lu*[1], *Ziyi He*[1], *Danning Wang*[1], *Xing Li*[1], *Guoshuai Zhu*[1], *Baojun Li*[1*], and *Hongbao Xin*[1*]

[1]Guangdong Provincial Key Laboratory of Nanophotonic Manipulation, Institute of Nanophotonics, Jinan University, Guangzhou 511443, China

Email: baojunli@jnu.edu.cn, hongbaoxin@jnu.edu.cn

Table of Contents

Figure S1. PTB characterization

Figure S2. Experimental setup

Figure S3. Characterization of the OHD rotation

Figure S4. Geometry of OHD in Simulation

Figure S5. Effect of Reynolds number changing on the removal of nano-biothreats

Figure S6. Comparison of removal efficiency for OHD and annularly scanning optical tweezers

Figure S7. Removal rate comparison

Figure S8. Comparison of removal efficiency for spheroidal algae and spindle diatoms

Figure S9. Observation of adenovirus

Figure S10. Collection and removal of 100-nm PS particles

Figure S11. Collection and removal of 800-nm PS particles

Figure S12. Collection and removal of 2-μm PS particles

Figure S13. Effect of pathogenic bacteria contamination on mammalian cells

Figure S14. Observation of *E. coli*

Figure S15. Collection and removal of *S. aureus*

Figure S16. Collection and removal of fluorescent *E. coli*

Figure S17. Effect of mycoplasma contamination on mammalian cells

Figure S18. Observation of Mycoplasmas

Figure S19. Effects on cell viability after 24-hour OHD treatment in confluent cells

Figure S20. Activity of HeLa cells and HL-60 cells co-cultured with OHD for 24 hours

Figure S21. Removal efficiency of OHD for removal of pathogenic bacteria

Figure S22. OHD array for efficiency-enhanced removal

Figure S23. *E. coli* removal using different OHD arrays

Figure S24. Antibacterial activity of chi-OHD

Figure S25. Biocompatibility test of chi-OHD

List of Supporting Movies

Movie S1: Optical control of OHD rotation.

Movie S2: Simulation comparation about the removal efficiency of OT and OHD.

Movie S3: Controllable collection and removal of adenoviruses.

Movie S4: Controllable collection and removal of pathogenic bacteria.

Movie S5: Controllable collection and removal of mycoplasmas.

Movie S6: Comparison of the removal efficiency using different OHD arrays.

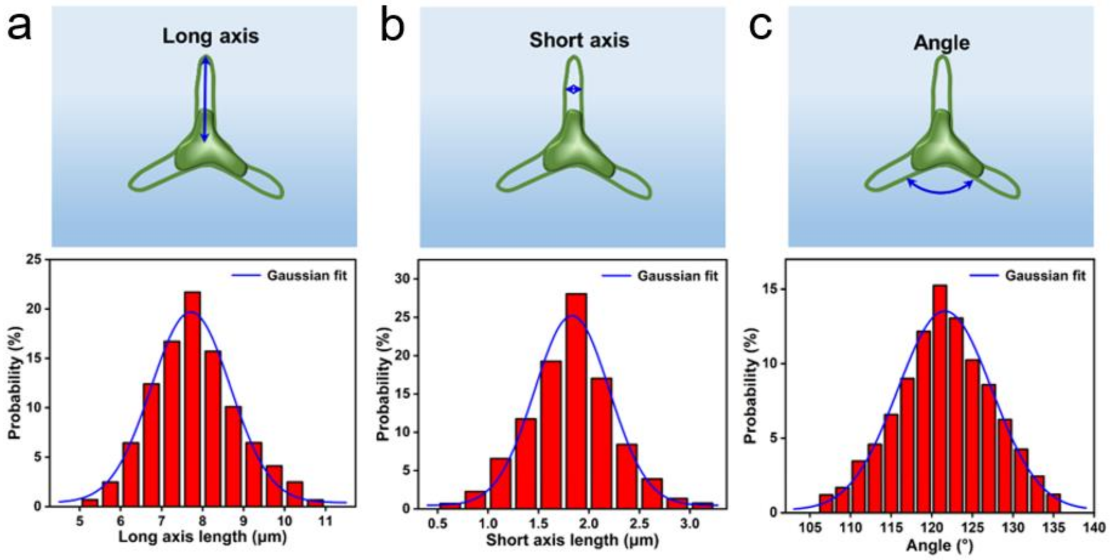

Figure S1. Size characterization of PTB cells based on over 100 cells repeat. Upper panels: schematic illustration of the PTB structure. Lower panels: histogram of size characterization with Gaussian fit. a) PTB long axis, the peak value of the length is 7.9 μm. b) PTB short axis, the peak value of the length is 1.8 μm. c) PTB arm angle, the peak value of the length is 120°.

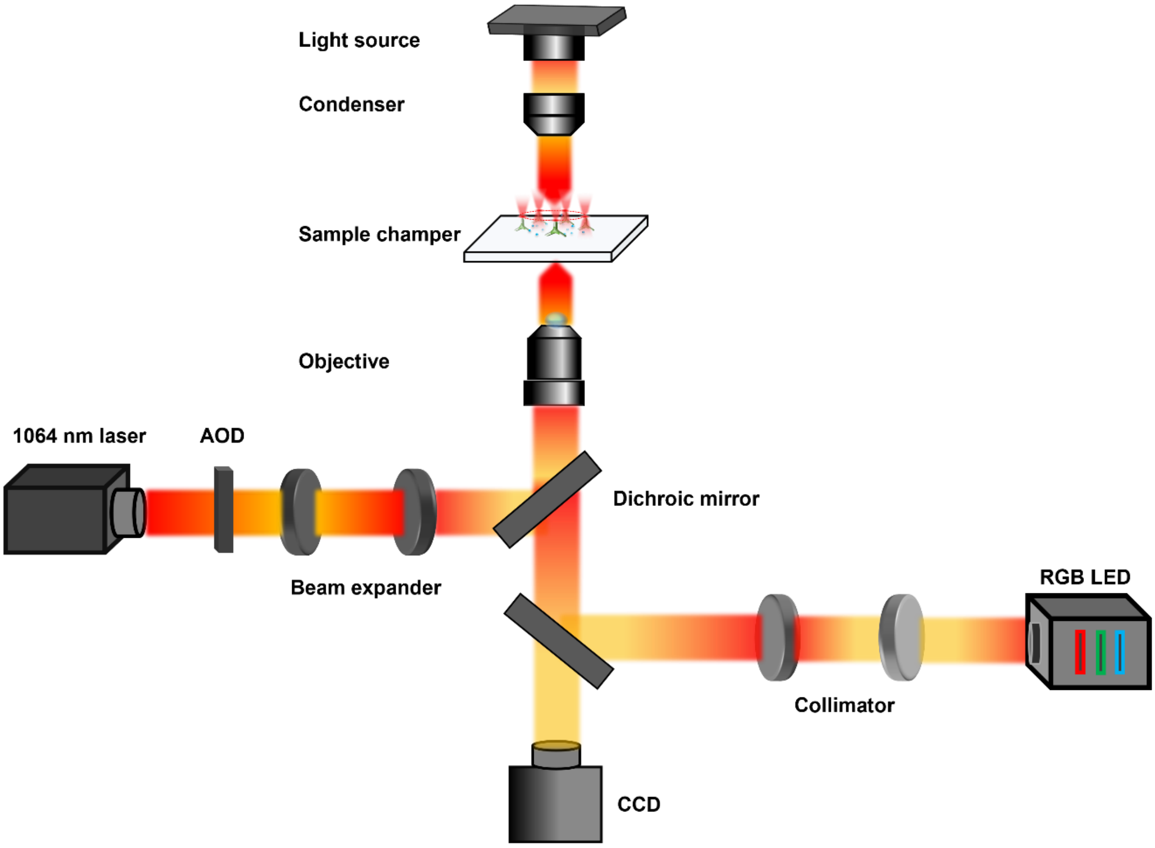

Figure S2. Schematic illustration of the experimental setup. The experimental setup consists of an inverted microscope (both fluorescence and bright-field) and a scanning optical tweezers system. The laser beam (wavelength: 1064 nm) is output from a continuous-wave solid-state laser source and focused on the sample plane through a 60× water-immersion inverted objective. For bright-field imaging, a halogen light source is used to illuminate the sample from the top. For fluorescence imaging, excitation light generated with an LED transmitted through a dichroic mirror is concentrated by an objective to illuminate the sample. The final experimental process can be captured by a high-speed CCD camera interfaced with a personal computer.

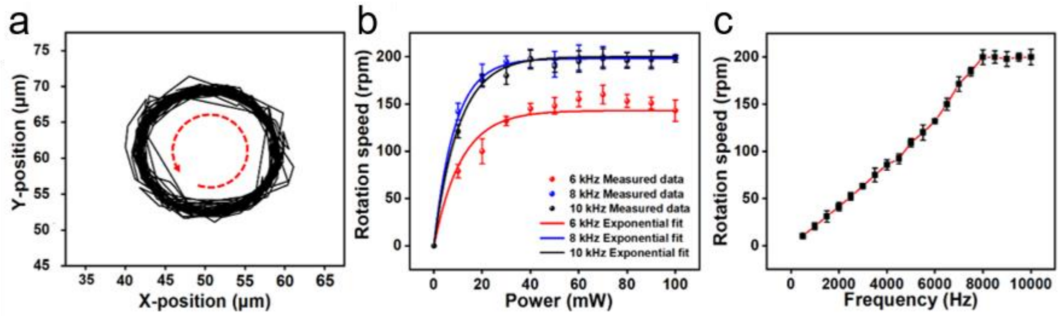

Figure S3. Characterization of the OHD rotation. a) Rotation trajectory of a OHD after being trapped and rotated by the annularly scanning optical trap (power: 50 mW, frequency: 8 kHz). The red circular arrow represents the counterclockwise (CCW) rotation. b) Measured rotation speed as a function of trapping power for different scanning frequency. c) Measured rotation speed as a function of scanning frequency at an optical power of 50 mW.

Simulation method

Overall: The flow field and optical field were simulated using the software COMSOL Multiphysics with a finite element method. The simulation mainly involves the following modules: "Laminar Flow Module (spf)", "Particle Tracing Module (fpt)", "RF Module (emw)" and the Multiphysics coupling between them. The parameters utilized in the simulation were consistent with those in the experiment.

Fluidic dynamic simulation: The geometric structure of OHD in simulation is shown in the Fig. S4, obtained by referring to the SEM image (Figure. 1b). The circular motion of OHD has a rotation radius (R) of 10 μm and a rotation frequency (w) of 200 rpm. The flow field around the OHD was obtained and analyzed through “Laminar Flow Module” of COMSOL Multiphysics.

Particle tracking: Particles behaviors under the synergy of OHD and optical force were simulated by coupling with the "Laminar Flow Module" and "Particle Tracking Module" of COMSOL Multiphysics. The parameters of the particles are as follows: 2 μm in size, 2000 kg/m3 in density, and randomly distributed in the fluid. The following formula was used to directly calculate the fluid force acting on the microspheres:[1]

$$F = 3\pi\mu\alpha v \quad (1)$$

where μ is dynamic viscosity, α is particle size, and v is the velocity of the particle. The values obtained by this method are basically consistent with the simulation results.

Optical field simulation: Optical field was simulated through the “RF Module” of COMSOL Multiphysics. The parameters are consistent with the experiment: wavelength 1064 nm, laser power 50 mW, and refractive index 1.45 of OHD. The optical force acting on the OHD and particles can be further obtained by integrating the Maxwell stress tensor ($<\mathbf{T}_M>$) along the surface.[2, 3]

$$<\mathbf{T}_M>=\mathbf{DE}^*+\mathbf{HB}^*-\frac{1}{2}(\mathbf{D}.\mathbf{E}^*+\mathbf{H}.\mathbf{B}^*)\mathbf{I} \quad (2)$$

where **D** and **H** are the electric displacement and magnetic field, respectively; **E*** and **B*** are the complex conjugates of the electric field **E** and magnetic flux field **B**, respectively; and I is the isotropic tensor. $\mathbf{F}_O$ can be expressed as:

$$\mathbf{F}_O = \oint_S \left(<\mathbf{T}_M>.\mathbf{n}\right) \mathrm{d}S \quad (3)$$

where **n** is the surface normal vector.

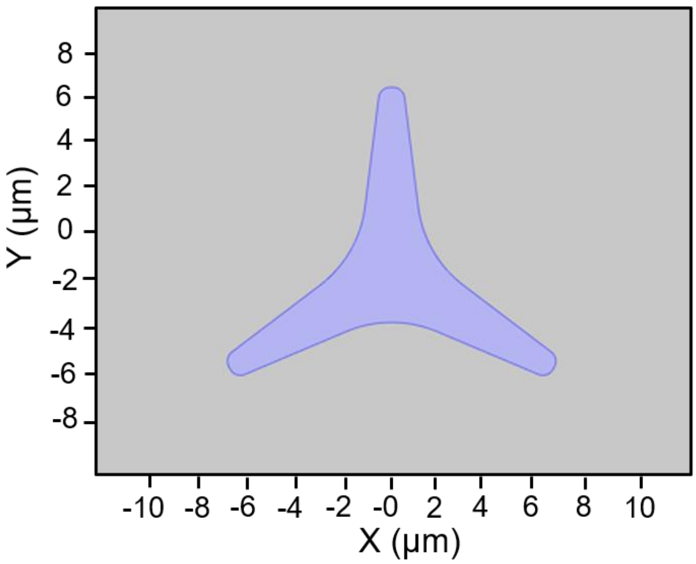

Figure S4. The OHD geometry used in the simulation.

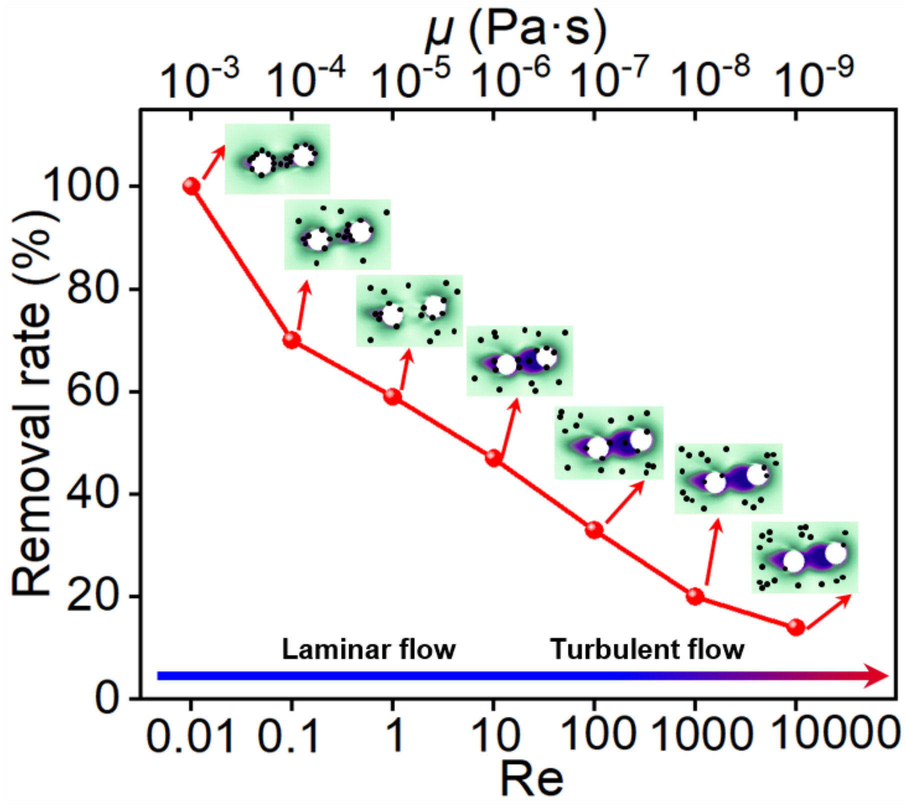

Figure S5. Removal rate of nano-biothreats as a function of Reynolds numbers

Comparison of removal efficiency for OHD and annularly scanning optical tweezers

The OHD exhibits a high-efficiency removal ability through opto-hydrodynamic force. As shown in Supporting Information Figure S6, under the same removal condition (removal time 16 s, scanning optical trapping power: 50 mW, scanning frequency: 8 kHz), the removal efficiency of the annular scanning optical tweezers is only about 20% (Figure S6a, Supporting Information), while that for OHD is 100% (Figure S6b, Supporting Information).

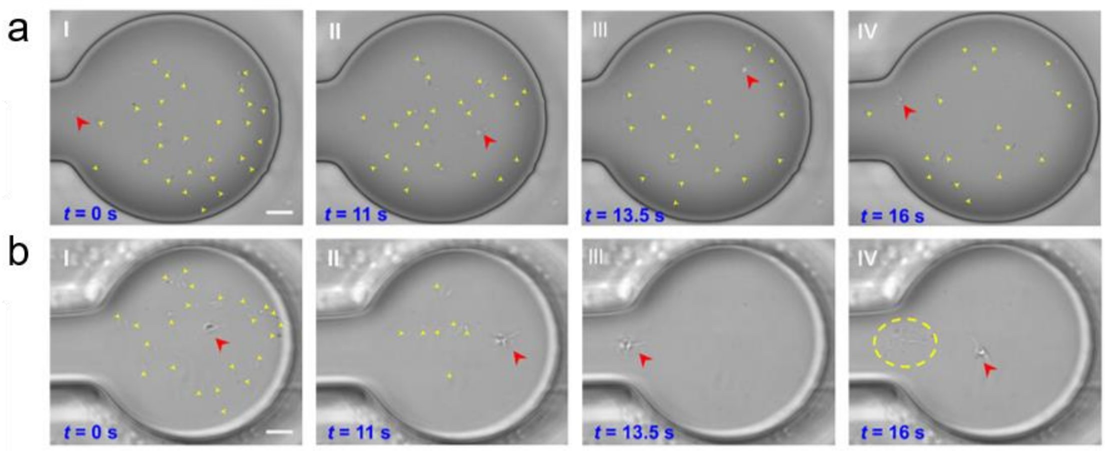

Figure S6. Bacteria removal using scanning optical tweezers and OHD. a) Removal of *E. coli* using scanning optical tweezers. Yellow and red arrows indicate *E. coli* and OHD, respectively. b) Removal of *E. coli* using OHD. Scale bars: 5 µm.

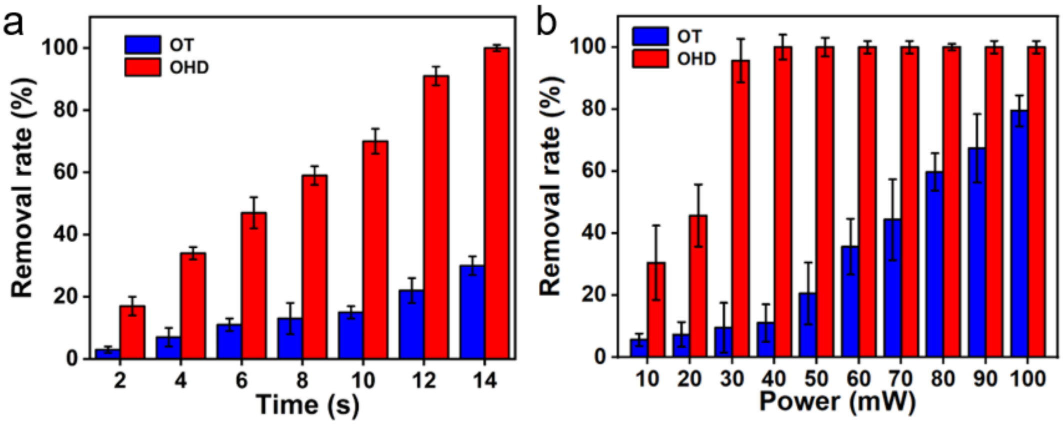

Figure S7. Removal rate comparison. a) Removal rate of the annular scanning optical tweezers and OHD at different time. b) Removal rate of the annular scanning optical tweezers and OHD with different power.

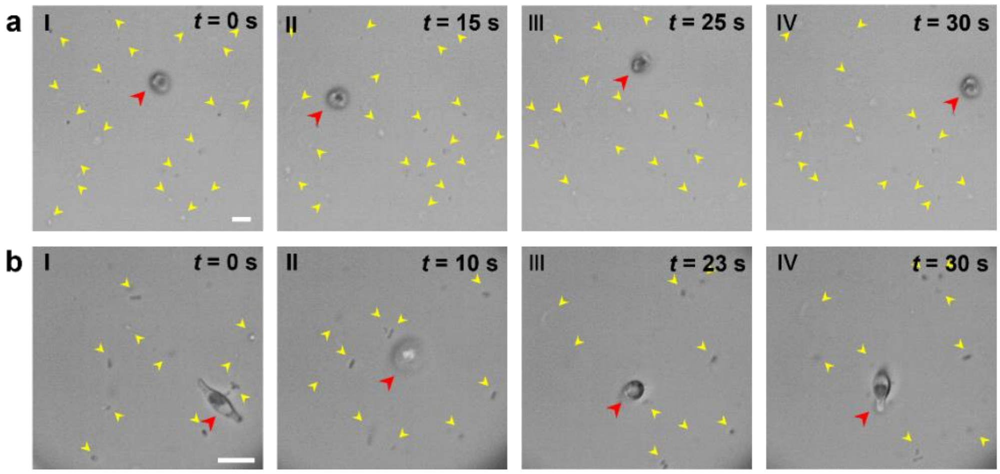

Figure S8. *E. coli* collection using (a) spherical algae and (b) spindle diatom, red arrows indicate the algae or diatom, and yellow arrows indicate the *E. coli*.

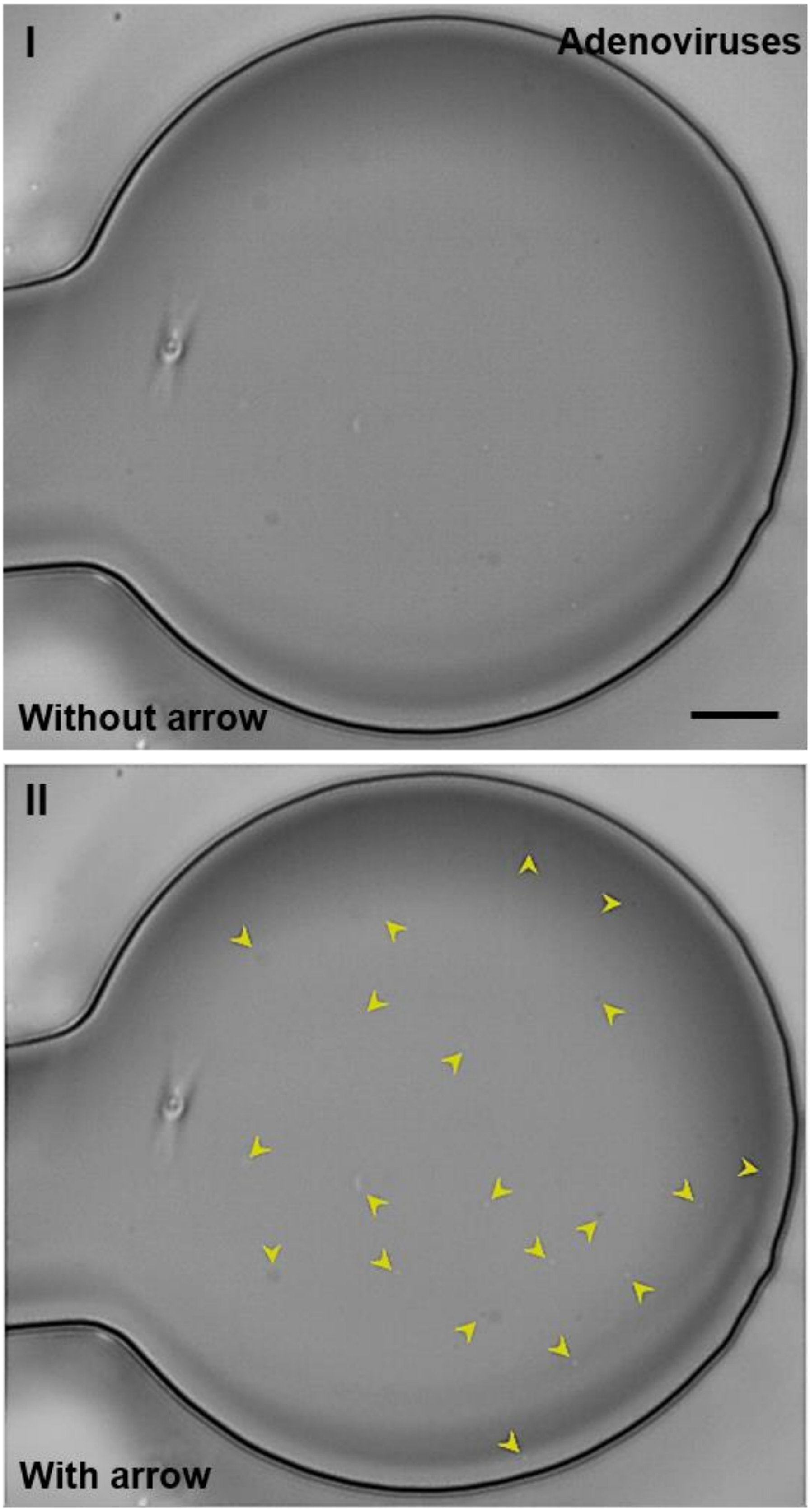

Figure S9. Adenovirus observation in (I) raw image, and (II) image with yellow arrow marked.

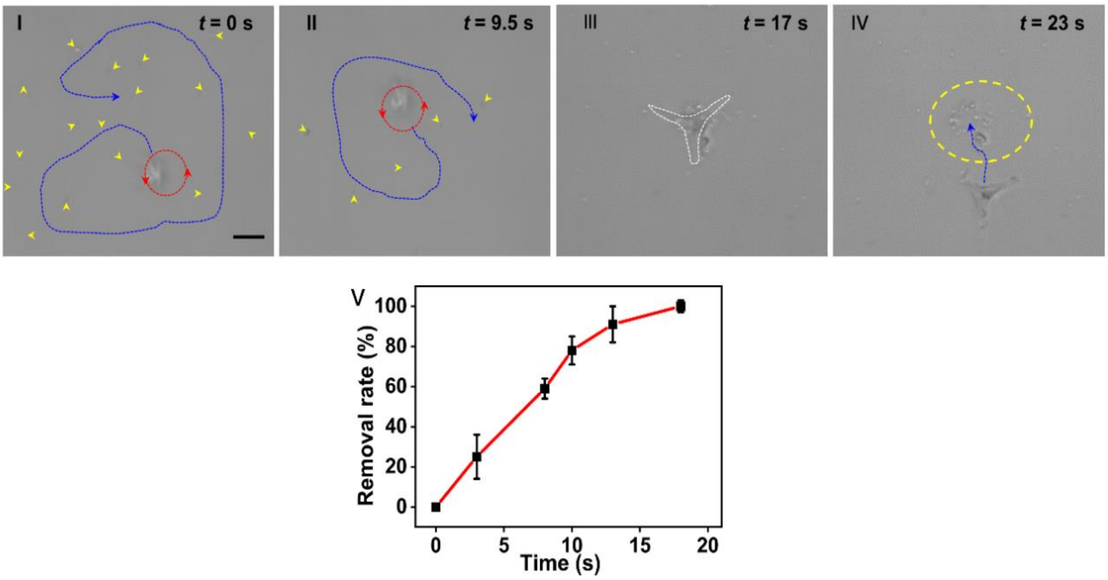

Figure S10. Controlled collection and removal of 100-nm PS particles by OHD. Panels I-IV are microscopic images of the collection and removal. Yellow arrows indicate the 100-nm PS particles, red circular arrows indicate the rotation direction of the OHD. The blue dotted lines indicate the motion trajectory of the OHD, and the white and yellow dotted boxes indicate the OHD and the final collected 100-nm particles, respectively. Panel V shows the removal rate as a function of time. Scale bars: 3 μm.

It should be noted that the above 100% removal rate is only for nanoparticles suspended in liquid. For the final observation of the collected nanoparticles after the collection process, the OHD is moved and placed on the surface of the glass slide by optical tweezers. On the surface of the glass slide, there are some nanoparticles firmly adhere to the slide surface. Such particles are not suspended in the liquid and therefore are not collected and removed by the OHD. Therefore, on panel IV of Figure S10, we can still observe some nanoparticles around.

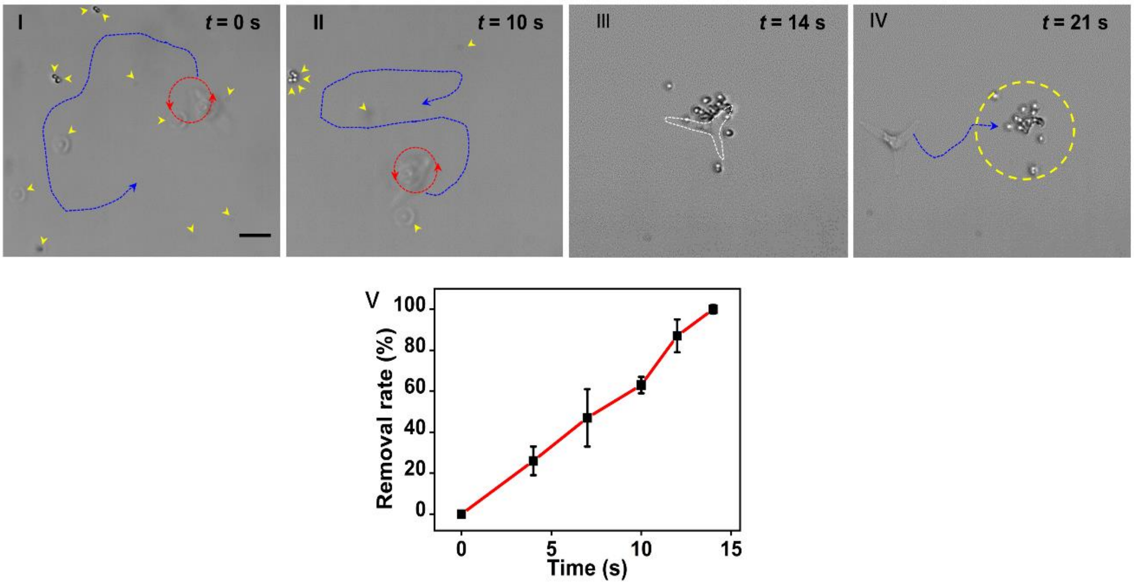

Figure S11. Controlled collection and removal of 800-nm PS particles. Panels I-IV are microscopic images of the collection and removal. Yellow arrows indicate the 800-nm PS particles, red circular arrows indicate the rotation direction of the OHD. The blue dotted lines indicate the motion trajectory of the OHD, and the white and yellow dotted boxes indicate the OHD and the final collected 800-nm particles, respectively. Panel V shows the removal rate as a function of time. Scale bars: 3 μm.

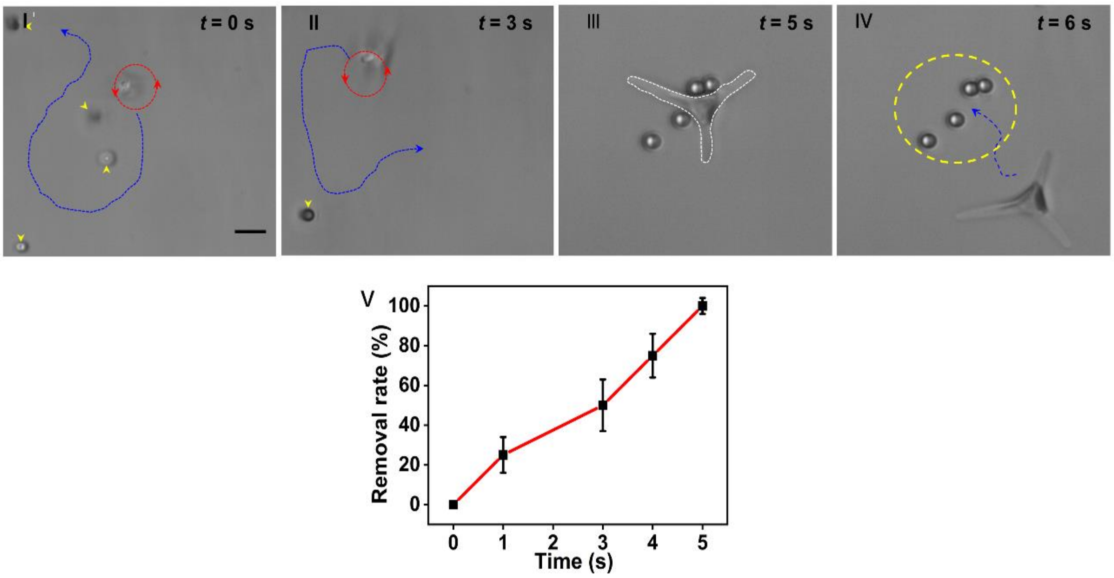

Figure S12. Controlled collection and removal of 2-µm PS particles. Panels I-IV are microscopic images of the collection and removal. Yellow arrows indicate the 2-µm PS particles, red circular arrows indicate the rotation direction of the OHD. The blue dotted lines indicate the motion trajectory of the OHD, and the white and yellow dotted boxes indicate the OHD and the final collected 2-µm particles, respectively. Panel V shows the removal rate as a function of time. Scale bars: 3 µm.

Effect of pathogenic bacteria contamination on mammalian cells

Due to the rapid bacterial reproduction in the cell culture medium, contamination of a small number of pathogenic *E. coli* can result in the death of both HeLa cells and HL-60 cells within 12 h (Figure S13, Supporting Information).

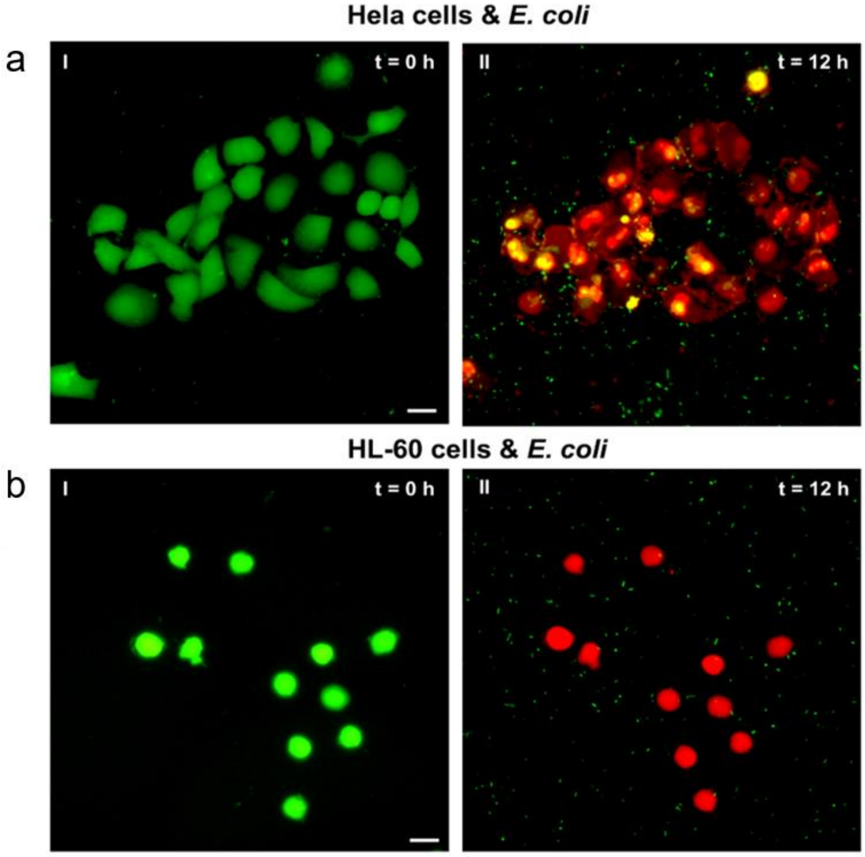

Figure S13. Effect of pathogenic bacteria on mammalian cell viability. a) Fluorescence images showing the viability of HeLa cells contaminated with *E. coli*. I, At the early stage of the contamination, there is only a few (about 25 *E. coli*) mixed with the HeLa cells. II, after contamination of 12 h, the number of *E. coli* is greatly enhanced (about 1.5×10^3), and the HeLa cells are dead due to the invasion of the *E. coli*. b) Fluorescence images showing the viability of HL-60 cells contaminated with *E. coli*. HL-60 cells are dead after 12-h of *E. coli* invasion. Green fluorescence indicates good cell viability and live *E. coli*. Red fluorescence indicates dead cells. Scale bars: 20 μm.

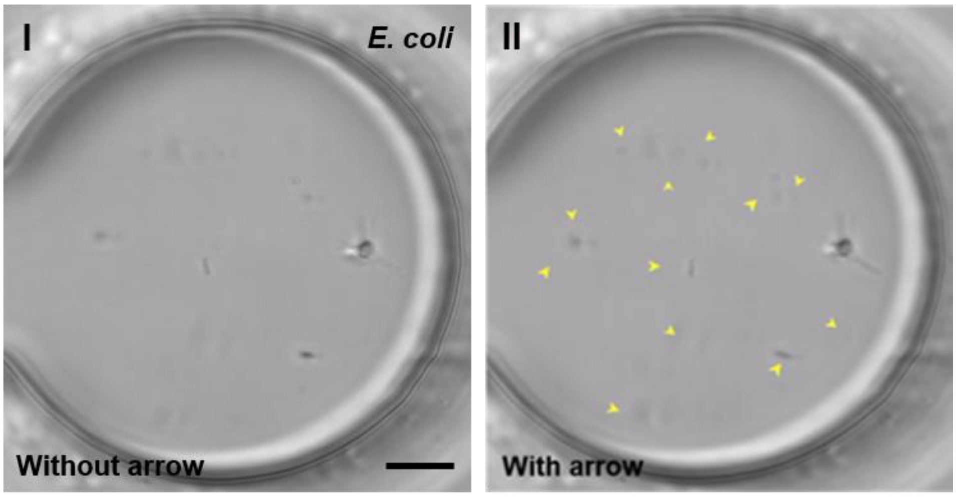

Figure S14. *E. coli*. observation in (I) raw image, and (II) image with yellow arrow marked.

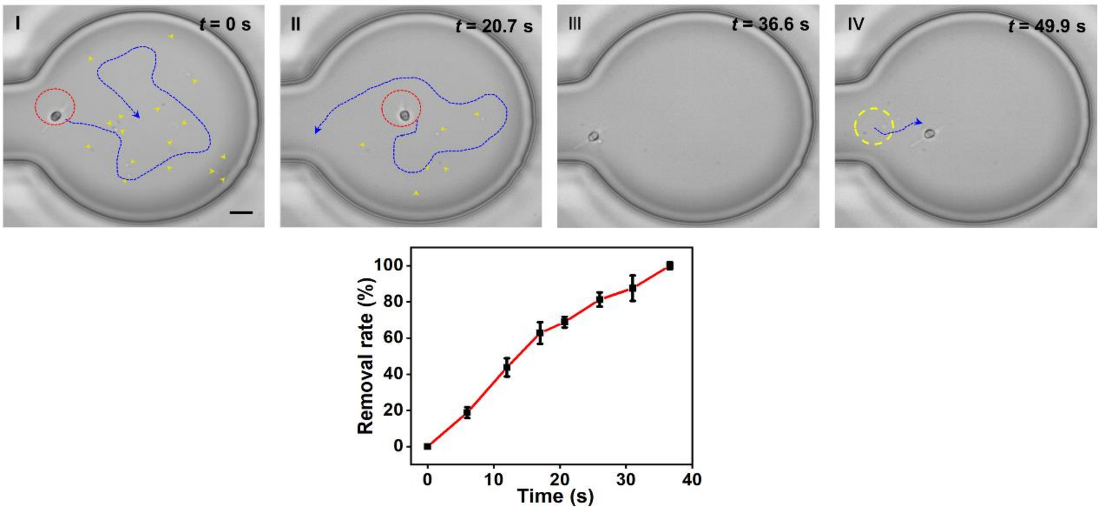

Figure S15. Controlled collection and removal of *S. aureus*. Panels I-IV are microscopic images of the collection and removal. Yellow arrows indicate the *S. aureus*, red circular arrows indicate the rotation direction of the OHD. The blue dotted lines indicate the motion trajectory of the OHD, and yellow dotted boxes indicate the OHD and the final collected *S. aureus*, respectively. Panel V shows the removal rate as a function of time. Scale bars: 3 µm.

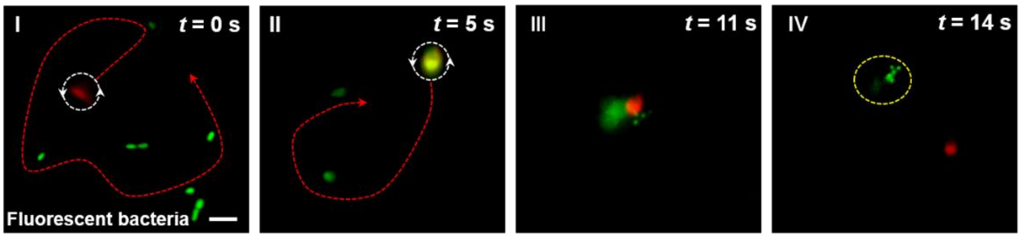

Figure S16. Collection and removal of fluorescent *E. coli* (green) using OHD (red). White circular arrows indicate the rotation direction of the OHD. The red dotted curves indicate the motion trajectory of the OHD. In panel IV, the yellow dotted circle indicates the final released *E. coli*. Scale bars: 2 μm.

Effect of mycoplasma contamination on mammalian cells

Similarly, when cells are contaminated with mycoplasmas, vacuolization of contaminated cells can be observed, with small black dots appeared around the cells. And eventually the cells are dead, as shown in Supporting Information Figure S17. When cells were stained with 4',6-Diamidino-2'-phenylindole (DAPI), in addition to the normal blue nuclei observed, a large amount of flocculent structures in the cytoplasm were also stained blue (Figure S17a, Supporting Information). However, in cells which were not contaminated with mycoplasma, only blue nuclei were observed (Figure S17b, Supporting Information).

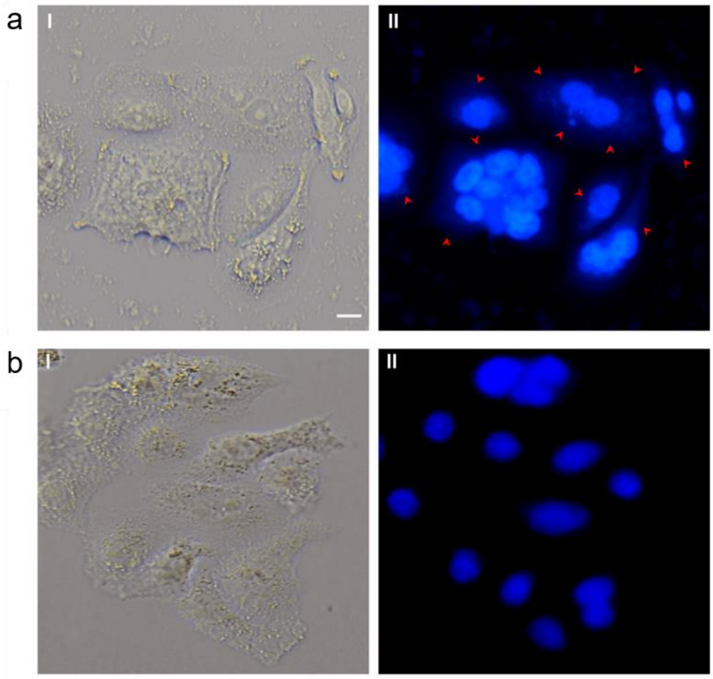

Figure S17. Effect of mycoplasma contamination on HeLa cells. a) Bright-field and corresponding fluorescence images showing cells contaminated with mycoplasma, respectively, and red arrows in the fluorescent image indicate mycoplasma. b) Bright-field and corresponding fluorescence images showing cells uncontaminated by mycoplasma, respectively. Scale bars: 10 μm.

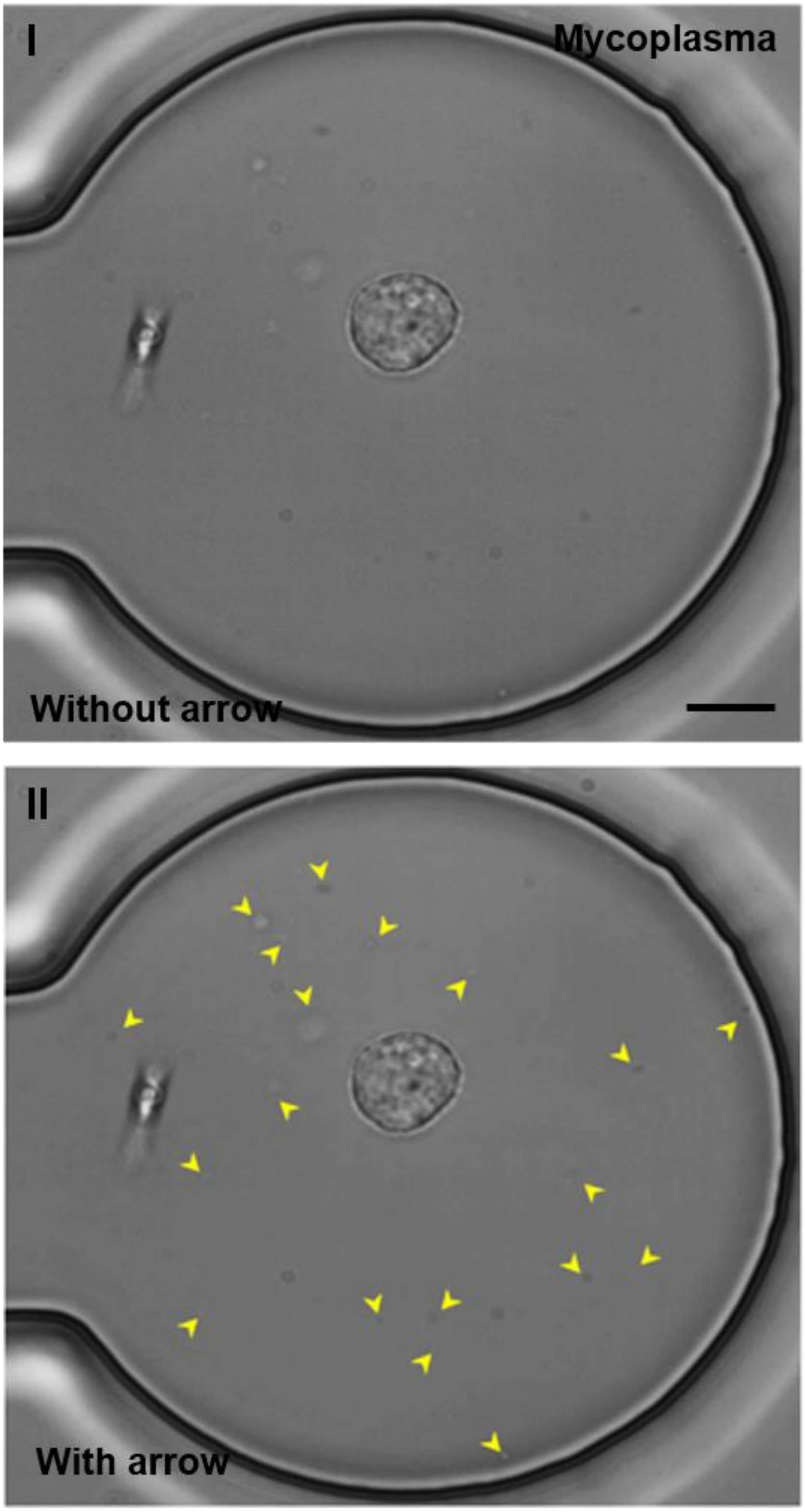

Figure S18. Mycoplasmas observation in (I) raw image, and (II) image with yellow arrow marked.

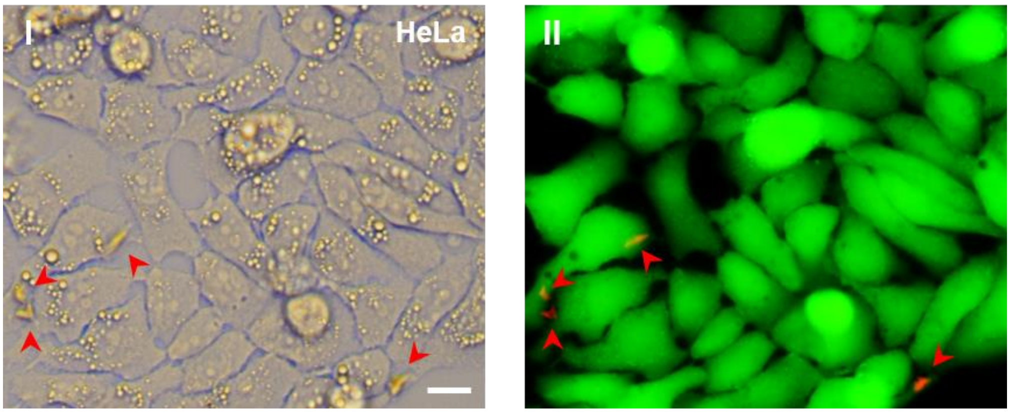

Figure S19. Effects on cell viability after 24-hour OHD treatment in confluent cells. (I) Bright field image and (II) corresponding fluorescence image showing the culturing of confluent cells treated with OHD. Red arrows indicate OHD, green fluorescence for living cells, and red fluorescence for OHD. Scale bar: 10 µm.

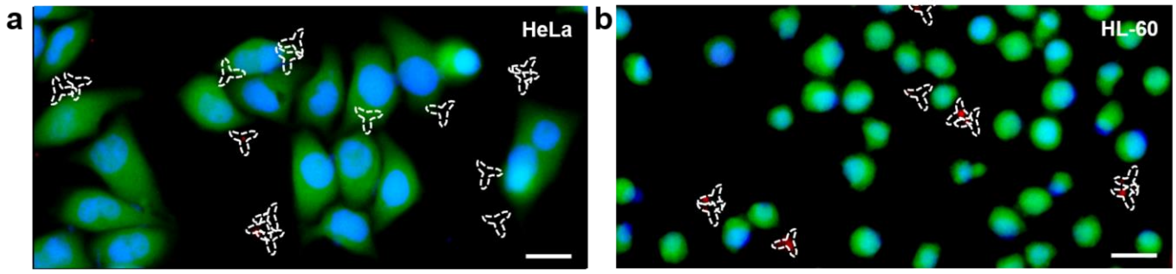

Figure S20. Fluorescence images showing (a) HeLa cells and (b) HL-60 cells co-cultured with OHD for 24 hours. White dashed triradiate structures indicate the position of OHD. Scale bars: 30 µm.

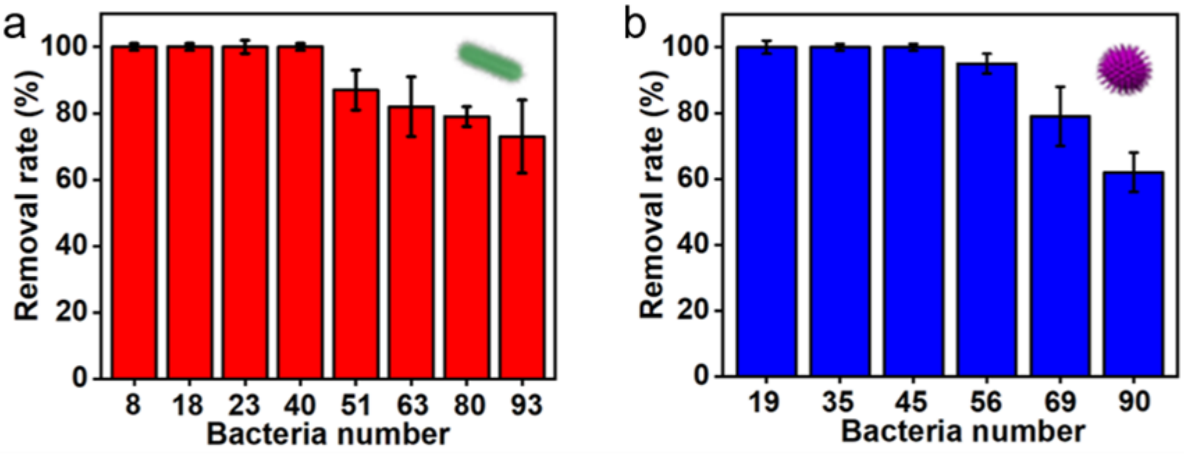

Figure S21. Controlled collection and removal of pathogenic bacteria. Removal rate of a) *E. coli* and b) *S. aureus* using a single OHD with different bacteria number in the channel.

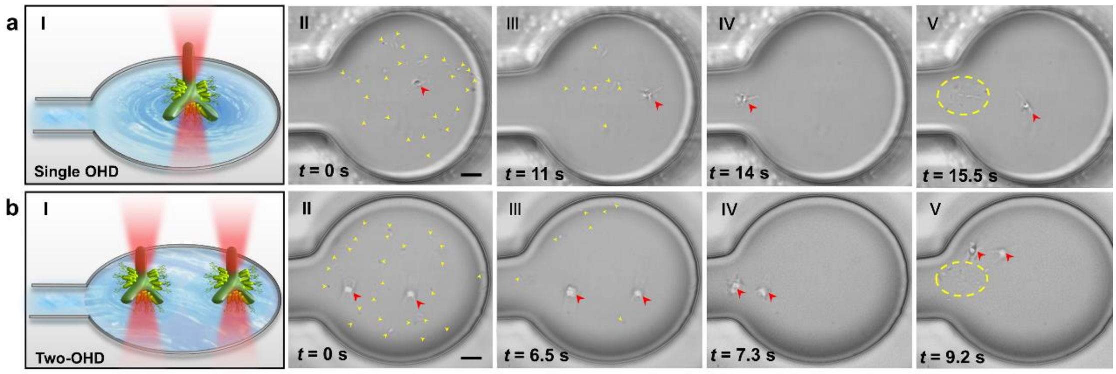

Figure S22. a, b) *E. coli* removal via a) a single OHD b) a two-OHD array. Panel I shows the schematic for removal, panels II-V show microscopic images of removal at different time. The yellow and red arrows indicate *E. coli* and OHD, respectively. The yellow dashed circle indicates the removed and released *E. coli*.

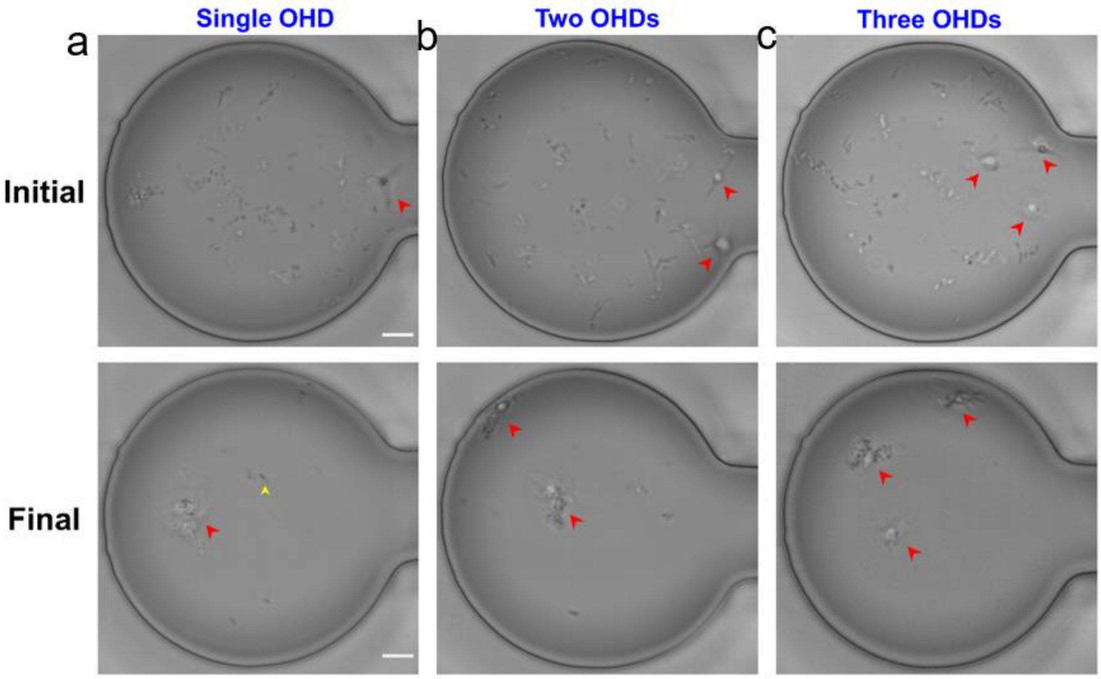

Figure S23. *E. coli* removal using a) single OHD, b) two-OHD array, and c) three-OHD array. Upper panels: initial state of the removal, lower panels: final state of the removal after 25 s. Red arrows indicate the OHD, yellow arrows in the lower panels indicate the *E. coli* that cannot be removed with a single OHD or two-OHD array. Scale bars: 2 µm.

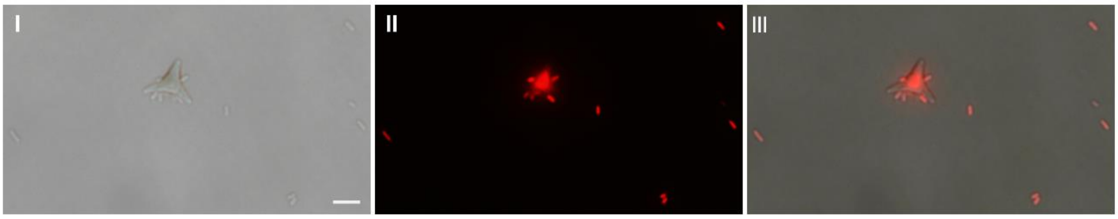

Figure S24. Antibacterial activity of chi-OHD. (I) Bright-field image, (II) Fluorescence image, (III) Merged image. Red fluorescence indicates chi-OHD and dead *E. coli*. Scale bars: 5 μm.

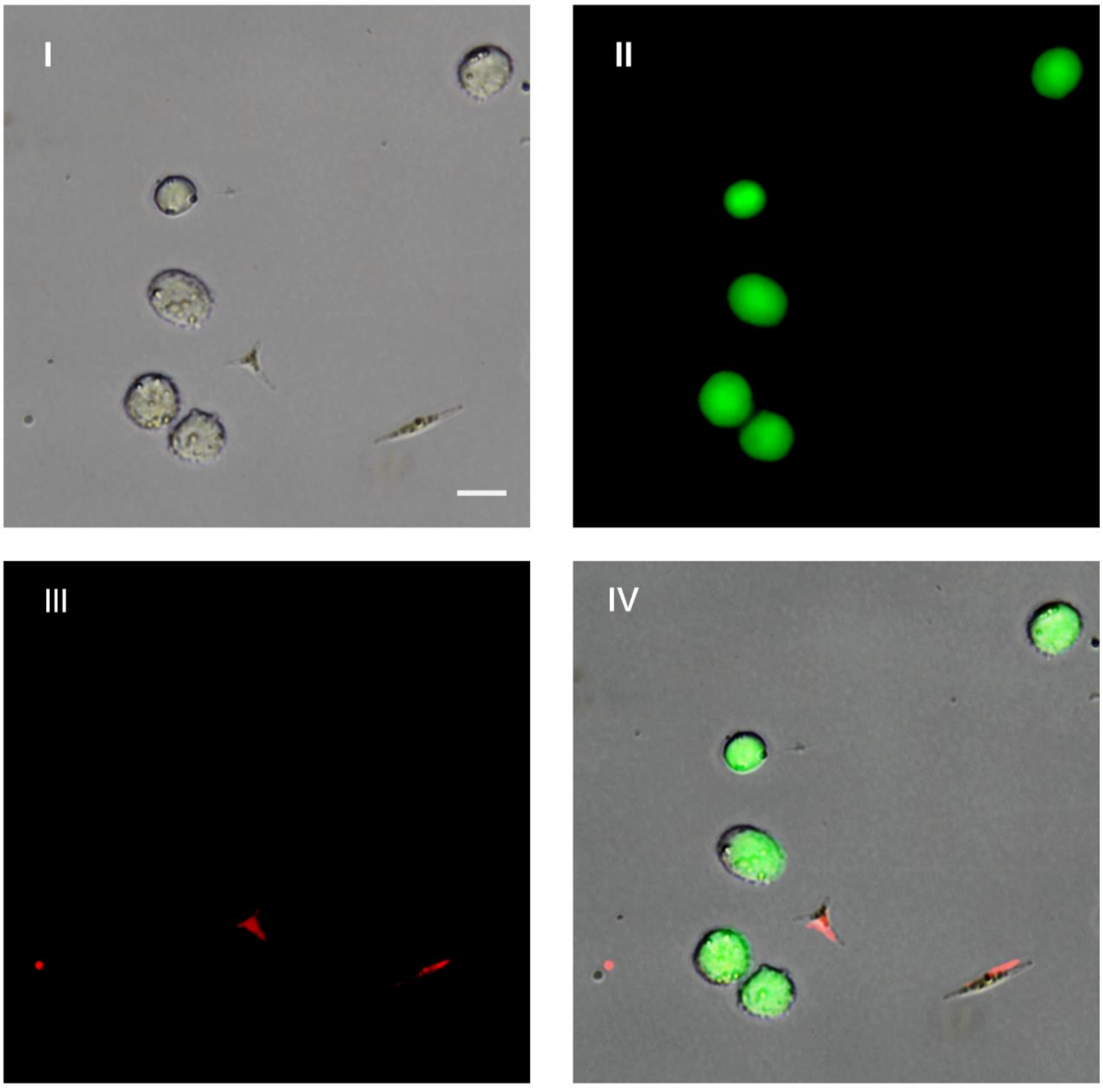

Figure S25. Co-culturing of chi-OHD with HL-60 cells for 1 day. (I) Bright-field image showing the chi-OHD with HL-60 cells. (II) Fluorescent image showing the HL-60 cells in good viability (green fluorescence for live cells). (III) Fluorescent image showing chi-OHD (red fluorescence for OHD). (IV) Merged image showing the chi-OHD and HL-60 cells. Scale bars: 10 μm.

References:

1. G. Stabile, G. Rozza, *Comput. Fluids* **2018**, 173, 273.
2. B. S. Schmidt, A. H. J. Yang, D. Erickson, M. Lipson, *Opt. Express* **2007**, 15, 14322.
3. L. Li, H. Xin, H. Lei, B. Li, *Appl. Phys. Lett.* **2012**, 101, 074103.
